# Effects of seasonal variation on phytochemicals contributing to the antimalarial and antitrypanosomal activities of *Breonadia salicina* using a metabolomic approach

**DOI:** 10.1016/j.heliyon.2024.e24068

**Published:** 2024-01-13

**Authors:** Dorcas Tlhapi, Isaiah Ramaite, Chinedu Anokwuru, Teunis van Ree, Ntakadzeni Madala, Heinrich Hoppe

**Affiliations:** a Department of Chemistry, Faculty of Science, Engineering and Agriculture, University of Venda, Private Bag X5050, Thohoyandou, 0950, South Africa; b Department of Basic Sciences, School of Science and Technology, Babcock University, Nigeria; c Department of Biochemistry, Faculty of Science, Engineering and Agriculture, University of Venda, Private Bag X5050, Thohoyandou, 0950, South Africa; d Department of Biochemistry and Microbiology, Rhodes University, Grahamstown, 6140, South Africa

**Keywords:** *Breonadia salicina*, Antimalarial activity, Antitrypanosomal activity, Phytochemicals, Chemical profile, Metabolomics, Chemometrics

## Abstract

This study involves the investigation of various plant parts of *Breonadia salicina* (Vahl) Hepper and J.R.I. Wood across multiple consecutive seasons. It aims to delve into the phytochemistry of these different plant parts and establish connections between the findings and their biological activities. This comprehensive approach employs metabolomics techniques, with the ultimate goal of exploring the potential for drug development. Samples were collected in Fondwe, a village in Limpopo (South Africa), based on local reports of the efficacy of this plant used by traditional healers in the area. The antimalarial and antitrypanosomal activities of samples collected over the seasons were determined with the parasite lactate dehydrogenase (pLDH) and specific *Trypanosoma brucei* assays, respectively. Consequently, a total of 24 compounds were tentatively identified through ultra-performance liquid chromatography with quadrupole time-of-flight mass spectrometry (UPLC-QTOF-MS). Chemical profiles of the different plant parts of *Breonadia salicina* collected in different seasons produced contrasting metabolic profiles. Chemometric analysis of the UPLC-QTOF-MS data enabled us to determine the chemical variability of the crude stem bark, root and leaf extracts (n = 48) collected over four consecutive seasons by evaluating the metabolomics fingerprinting of the samples using an untargeted approach. Principal component analysis (PCA), hierarchical cluster analysis (HCA), and partial least squares discriminant analysis (PLS-DA) indicated the existence of two key clusters that are linked to the root, stem bark, and leaves. The stem and root chemistry differed from that of the leaves. Seasonal variations were noted in each plant part, with autumn and winter samples closely grouped compared to spring and summer samples in the methanol leaf extracts. Biochemometric analysis could not relate specific compounds to the antimalarial and antitrypanosomal activities of the active extracts, underscoring the intricate interactions among the secondary metabolites. This study further confirms the optimal plant parts to collect in each season for the most effective antimalarial and antitrypanosomal activities.

## 1 Introduction

Seasonal change is one of several environmental conditions that affect the production of metabolites in plants [1,2]. Metabolites may be formed and stored over a period in response to seasonal change. Such variations in metabolite formation can result in prominent chemical changes in any plant material, affecting the value of the bioactive compounds [2]. Physiological activity, which relies on the presence of specific compounds, is subject to these variations; several studies have provided evidence of these variations [[1], [2], [3], [4], [5], [6], [7], [8], [9], [10]]. Plant growth can be affected by environmental factors such as temperature, height above mean sea level, rainfall, light intensity, soil humidity, and change of season [11,12].

Furthermore, the chemical composition, such as the concentration, availability and bioactivity determine the biological product. These changes in chemical profile of a plant could cause routine difficulties for the validation and standardization of its therapeutic value [13]. The medicinal value of a plant might be dismissed if it lacks activity, yet this judgment often overlooks factors influencing the production of phytochemicals. The traditional herbal market needs standardized plant material with ubiquitous active secondary metabolites, which ultimately translate to less variation in pharmacological activity [14]. Therefore, to maintain quality, continuous observation of the composition and pharmacological activity in response to environmental conditions affecting the production of phytochemicals is crucial for production of marketable traditional plant material. Various analytical approaches can be used to study the effects of environmental factors such as seasons on the production of phytochemicals, for example metabolomics in conjunction with multivariate data analysis [15]. Plant metabolomics can distinguish such variations in plant metabolites caused by different environmental factors [16]. A few studies have provided evidence of the success of the metabolomics technique in exploring the influence of seasons on the phytochemical composition of significant medicinal plants [14,[17], [18], [19], [20]]. Ethnobotanical studies have revealed that *Breonadia salicina* is commonly used for the treatment of malaria and trypanosomiasis [21,22], indicating that the plant produces compounds with antimalarial and antitrypanosomal activities. However, there are no reports correlating the phytochemistry to the antimalarial and antitrypanosomal activities of this plant. The traditional approach to phytochemistry often involves bioactivity guided fractionation and most often the minor, but active, constituents are lost in this process. The reductionist approach of attributing the activity of a plant to a few compounds does not provide comprehensive insight into the complexity of the interaction of plants with the environment [23]. In recent times, metabolomics approaches have unravelled previous challenges in phytochemistry. For example, phytochemical studies of *B. salicina* have yielded compounds such as *α*-amyrin, ursolic acid, and lupeol; 2,4-dihydroxycinnamic acid and stigmasterol; coumarins like 7-hydroxycoumarin, 6-hydroxy-7-methoxycoumarin, and 7-(*β*-d-apiofuranosyl (1–6)-*β*-d-glucopyranosyl) umbelliferone; saturated fatty acids (hexadecane and palmitic acid), a disaccharide (sucrose), an oleanane triterpenoid saponin (bodinioside Q), a monosaccharide (d-galactopyranose), a flavonoid (kaempferol 3-*O*-(2ʹʹ-*O*-galloyl)-glucuronide) and chlorogenic acid (5-*O-*caffeoylquinic acid) using a reductionist approach [[24], [25], [26], [27]]. Although isolation of phytochemicals from *B. salicina* plant parts has been previously reported [[24], [25], [26]], not much has been disclosed of isolation techniques used. The current work shows that the metabolomics approach is a superior method compared to reductionist, classical or traditional approaches. Furthermore, reductionist and classical approaches are time-consuming and often inefficient in the exploration of chemical constituents, unlike the metabolomics approach. Therefore, there is a need to evaluate any possible variation in chemical constituents contributing to the biological activities.

A few studies have revealed the presence of a broad range of phytochemicals in the Rubiaceae family [28]. However, the influence of the seasons on the metabolites and bioactivity of *Breonadia salicina* has not yet been studied. Due to its use in traditional medicine, it is necessary to explore the effect of seasonal variation on its biological activities. The chemical profiles of the root, stem bark, and leaf samples, collected in autumn, winter, summer and spring, were explored using LC-MS-based metabolomics approach. Furthermore, the chemical variability of the various plant parts of *B*. *salicina*, collected during the four seasons, was studied by chemometrics since reductionist, traditional and classical methods are inadequate to explore the distribution of the chemical constituents in the various plant parts of *B. salicina* and identify the bioactive antimalarial and antitrypanosomal constituents. Therefore, this study was aimed at metabolomic exploration of seasonal variations on the chemical composition and the antimalarial and antitrypanosomal activities of various plant parts of *B. salicina*.

## 2 Experimental

### 2.1 Sampling and extraction of seasonal samples

**Fig. 1:**
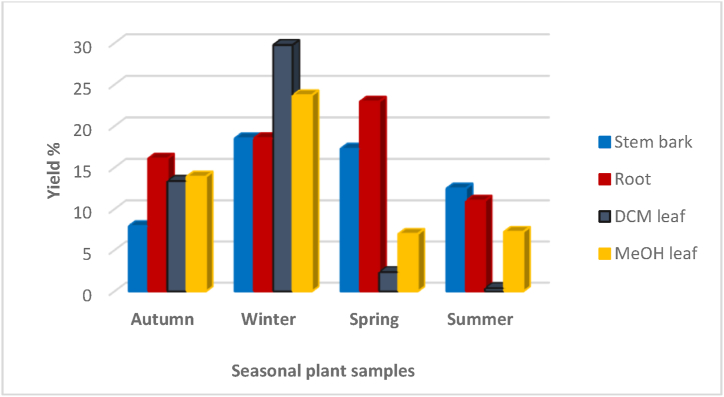
Percentage yields of seasonal plant samples of *B. salicina.*

The different plant parts were collected at Fondwe, a village in the South African province of Limpopo (22°55*′*31.9*″* S, 30°15*′*45.0*″* E) during autumn (March 2020), winter (July 2020), spring (October 2020) and summer (December 2020). A specimen with voucher number BD 001–012 was placed in the University of Venda herbarium. After four weeks' air-drying, the plant parts were ground using an industrial mill (NETZSCH, Selb, Germany). About 2 L methanol was added to 1 kg each of stem and root samples, and after soaking at ambient temperature for 48 h, the supernatants were filtered and then evaporated at 45 °C to yield dried extracts, as shown in Fig. 1. The leaf samples were extracted at room temperature for 48 h with 2 L dichloromethane followed by 2 L methanol. As before, the supernatants were filtered and evaporated at 45 °C to obtain dried extracts, as shown in Fig. 1. The data for the seasonal samples and voucher numbers are provided in Table 1. A working solution (1 mg/mL) was prepared in HPLC grade methanol (Merck, Darmstadt, Germany) for UPLC-QTOF-MS analysis. Samples (n = 48) were prepared and analysed by LC-MS in triplicate (Table 2).

**Table 1:** Seasonal plant samples and voucher numbers of *B. salicina*.

Seasonal samples	Voucher Number	Dried samples (before extraction)	Dried extracts (after extraction)
**Autumn**			
stem bark	BD 001	1.05 kg	83.31 g
Root	BD 002	0.78 kg	125.451 g
Leaf	BD 003	0.98 kg	–
DCM leaf	–	–	131.281 g
MeOH leaf	–	–	136.33 g
**Winter**			
stem bark	BD 004	0.76 kg	140.75 g
Root	BD 005	0.29 kg	53.767 g
Leaf	BD 006	0.61 kg	–
DCM leaf	–	–	181.62 g
MeOH leaf	–	–	144.705 g
**Spring**			
stem bark	BD 007	0.75 kg	129.5 g
Root	BD 008	0.33 kg	75.74 g
Leaf	BD 009	0.62 kg	–
DCM leaf	–	–	14.59 g
MeOH leaf	–	–	43.26 g
**Summer**			
stem bark	BD 010	1.25 kg	156.022 g
Root	BD 011	0.88 kg	96.93 g
Leaf	BD 012	0.32 kg	–
DCM leaf	–	–	1.249 g
MeOH leaf	–	–	23.155 g

**Table 2:** Seasonal plant samples and codes of *B. salicina* in triplicates (n = 48).

Seasonal samples	Codes
**Autumn**	
Stem bark	Sa1, Sa2 and Sa3
Root	Ra1, Ra2 and Ra3
DCM leaf	Lda 1, Lda 2 and Lda 3
MeOH leaf	Lma1, Lma2 and Lma3
**Winter**	
Stem bark	Sb1, Sb2 and Sb3
Root	Rb1, Rb2 and Rb3
DCM leaf	Ldb1, Ldb2 and Ldb3
MeOH leaf	Lmb1, Lmb1 and Lmb1
**Spring**	
Stem bark	Sc1, Sc2 and Sc3
Root	Rc1, Rc2 and Rc3
DCM leaf	Ldc1, Ldc1 and Ldc1
MeOH leaf	Lmc1, Lmc1 and Lmc1
**Summer**	
Stem bark	Sd1, Sd1 and Sd1
Root	Rd1, Rd1 and Rd1
DCM leaf	Ldd1, Ldd1 and Ldd1
MeOH leaf	Lmd1, Lmd1 and Lmd1

### 2.2 Antimalarial activity

*Plasmodium falciparum* (3D7 strain) malaria parasites (Manassas, VA, USA) were preserved as described before [27] with 2–4 % hematocrit human red blood cells. The seasonal crude extracts (50 μg/mL) were added in 96-well plates and incubated for 48 h in a 37 °C CO_2_ incubator. After incubation, 20 μL culture was transferred to a different clear 96-well plate with a concoction of 125 μL of Malstat and Nitroblue tetrazolium (NBT)/Phenazine ethosulphate (PES) solutions [29], and the parasite lactate dehydrogenase (pLDH) enzyme was used to determine the activity of the measured solutions. The purple colour change was monitored at 620 nm (Abs_620_) in a Spectramax M3 microplate reader (Molecular Dynamics Inc., Chatsworth, CA, USA), and the absorbance at 620 nm (Abs_620_) was changed to percentage (%) parasite viability comparative to untreated control wells. For dose-response analysis, the experiments were executed as described above, but the parasite cultures were incubated in 96-well plates with threefold serial dilutions of the crude seasonal extracts, tested at 300 μg/mL. Graphs of the % parasite viability versus the log (sample concentration) were used to derive the IC_50_ values by non-linear regression using GraphPad Prism (GraphPad Software, San Diego, CA, USA).

### 2.3 Antitrypanosomal testing

To evaluate trypanocidal activity, the samples were added in triplicate to in vitro *T. b. brucei* cultures (strain Lister 427) in 96-well plates (2.4 × 10^4^ parasites/well) at 50 μg/mL for the seasonal crude extracts (50 μg/mL). The extracts with parasites were cultured in a medium based on Iscove's Modified Dulbecco's Medium, as reported before [30]. After 24 h incubation at 37 °C in a humidified 5 % CO_2_ incubator, resazurin (Sigma-Aldrich) was added to a final concentration of 0.05 mM and incubation was continued for a further 24 h, after which conversion of resazurin to resorufin was measured in a Spectramax M3 fluorescence plate reader (Molecular Devices; Exc 560/Em590). The % cell viability in wells containing test samples was calculated relative to untreated control wells using the fluorescence readings, after subtracting background fluorescence in wells without cells. For dose-response analysis, the experiments were executed as described above, but the parasite cultures were incubated in 96-well plates with threefold serial dilutions of the crude seasonal extracts tested at 300 μg/mL. The IC_50_ values were determined from the graph of percentage parasite viability versus log (sample concentration) using GraphPad Prism (GraphPad Software, San Diego, CA, USA).

### 2.4 UPLC-QTOF-MS analysis

A liquid chromatography-quadrupole time-of-flight tandem mass spectrometer (LC-MS-9030 q-TOF, Shimadzu Corporation, Kyoto, Japan) fitted with a Shim-pack Velox C_18_ column (100 mm × 2.1 mm with particle size 2.7 μm) was used. The oven temperature was 50 °C. The injection volume was 5 μL and the samples were separated over a 30 min binary solvent gradient. The flow rate was kept constant at 0.3 mL/min using a binary solvent mixture of 0.1 % formic acid in water (Eluent A) and 0.1 % formic acid in methanol (Eluent B). The gradient was gradually increased from 3 to 30 min to facilitate the separation of the compounds within the samples. Briefly, Eluent B was kept at 5 % from 0 to 3 min, gradually increased from 5 to 40 % between 3 and 8 min, and finally increased to 40–95 % between 8 and 23 min. Eluent B was then kept isocratic at 95 % between 23 and 25 min. The gradient was returned to original conditions of 5 % at 25–27 min, and re-equilibration at 5 % occurred at 27–30 min. The liquid chromatographic eluents were then subjected to a Quadruple Time-of-Flight high-definition mass spectrometer for analysis in negative ESI mode. The q-TOF-MS conditions were as follows: 400 °C heat block temperature, 250 °C Desolvation Line (DL) temperature, 42 °C flight tube temperature, and 3 L/min nebulization and dry gas flow.

### 2.5 Chemometric analysis of LC-MS data

The raw data of 48 seasonal samples provided by the UPLC-MS analysis were exported to Microsoft Excel [31] and then imported into MetaboAnalyst 5.0, a free metabolomics data analysis tool available online (www.metaboanalyst.ca/MetaboAnalyst/home.xhtml). Principal component analysis (PCA)**,** partial least squares discriminant analysis (PLS-DA) and a loading score plot were obtained with Pareto scaling method. Hierarchical cluster analysis (HCA) was carried out from the PCA model and the resulting dendrogram was used to evaluate sample groupings and trends in the *Breonadia salicina* dataset. An orthogonal projection to latent structures discriminant analysis (OPLS-DA) model was then constructed by assigning class identifiers to the samples. The loadings plot constructed from the OPLS-DA model was used to identify biomarkers.

### 2.6 Statistical analysis

For the antimalarial dose-response tests, parasites were cultured in threefold serial dilutions of the dichloromethane leaf extracts collected in autumn, winter, spring and summer. The IC_50_ values were derived from graphs of percentage viability versus log (sample concentration) calculated using non-linear regression (GraphPad Prism v.5.02). The standard antiplasmodial chloroquine was used as a positive control for antiplasmodial activity. For the antitrypanosomal dose-response tests, parasites were cultured in threefold serial dilutions of the methanol leaf extracts collected in autumn, spring and summer; and the dichloromethane leaf extracts collected in autumn, winter, spring and summer. The IC_50_ values were obtained using a non-linear regression application (GraphPad Prism v.5.02) by plotting viability against log (sample concentration). The positive control was the existing anti-trypanosomiasis drug pentamidine. The statistical differences of the samples in the antimalarial and antitrypanosomal tests were evaluated using one-way analysis of variance (ANOVA, GraphPad Prism 6); *p* < 0.05 was considered statistically significant.

## 3 Results and discussion

### 3.1 Constituents of the seasonal crude stem bark, root and leaf extracts identified by UPLC-QTOF mass spectrometry

Twenty-four *Breonadia salicina* metabolites were identified by UPLC-QTOF-MS analysis of the crude extracts of roots, stem bark, and leaves collected during the four seasons. The metabolites were identified tentatively by comparison of their MS data with literature values (Table 3). Peak 1 (Fig. S1) with precursor ion at *m/z* 533.17426 [*M* − H]^-^ and molecular formula C_19_H_34_O_17_ was identified as quinic acid diglucoside; this molecule produced a product ion at *m/z* 191.05626, which is similar to results reported elsewhere [32]. Peak 2 (Fig. S2) with precursor ion at *m/z* 353.08943 [*M* − H]^-^ and molecular formula C_16_H_18_O_9_ was identified as chlorogenic acid; this molecule produced a product ion at *m/z* 191.05672, similar to what has been reported elsewhere [33]. Peak 3 (Fig. S3) with precursor ion at *m/z* 390.11424 [*M* − H]^-^ and molecular formula C_20_H_22_O_8_ was identified as *trans*-resveratroloside; this molecule produced the following product ion at *m/z* 389.11129, and our results are similar to what has been reported elsewhere [34]. Peak 4 (Fig. S4) with precursor ion at *m/z* 403.12695 [*M* − H]^-^ and molecular formula C_17_H_24_O_11_ was identified as oleoside 11-methylester, this molecule produced the following product ion at *m/z* 404.13018, and our results are similar to what has been reported elsewhere [35]. Peak 5 (Fig. S5) with precursor ion at *m/z* 515.12236 [*M* − H]^-^ and molecular formula C_25_H_24_O_12_ was identified as 3,4−dicaffeoylquinic acid, this molecule produced the following product ion at *m/z* 353,191, and our results are similar to what has been reported elsewhere [36]. Peak 6 (Fig. S6) with precursor ion at *m/z* 537.16508 [*M* − H]^-^ and molecular formula C_32_H_26_O_8_ were identified as 4,8,4′,8′-tetramethoxy-[1,1′-biphenanthrene]-2,7,2′,7′-tetrol; our results are similar to what has been reported elsewhere [37]. However, peaks 7 (Rt = 12.670 min), 8 (Rt = 0.746 min), 9 (Rt = 8.735 min), and 10 (Rt = 0.760 min), with [*M* − H]^-^ ions at *m/z* 439.32578 (Fig. S7), *m/z* 369.08500 (Fig. S8), *m/z* 455.31978 (Fig. S9), and *m/z* 191.05725 (Fig. S10), respectively, were identified as pfaffic acid (C_29_H_44_O_3_), ferulic acid 4-*O*-glucuronide (C_16_H_18_O_10_), ursolic acid (C_30_H_48_O_3_), and quinic acid (C_7_H_12_O_6_), respectively [35,[38], [39], [40]]. Peak 8 generated fragment ions at *m/z* 161 and 353, whereas peaks 7, 9 and 10 did not show recognizable fragmentation patterns. Moreover, peak 11 (Rt = 5.218 min, C_16_H_22_O_11_) was identified unequivocally as deacetyl asperuloside acid (Fig. S11, *m/z* 389.11215 [*M* − H]^-^), peak 12 (Rt = 7.005, C_27_H_30_O_16_) as rutin (Fig. S12, *m/z* 609.15209 [*M* − H]^-^), and peak 13 (Rt = 7.290, C_25_H_24_O_12_) as di-*O*-caffeoylquinic acid (Fig. S13, *m/z* 515.12463 [*M* − H]^-^) [[40], [41], [42]]. In the MS-MS spectra of peaks 11, 12, and 13, molecular ions appeared at *m/z* 390.11531; *m/z* 463,447 and *m/z* 353.09154, respectively. These were followed by peak 14 (Fig. S14, *m/z* 327.22003 [*M* − H]^-^), peak 15 (Fig. S15, *m/z* 271.23033 [*M* − H]^-^), peak 16 (Fig. S16, *m/z* 490.36367 [*M* − H]^-^), peak 17 (Fig. S17, *m/z* 433.13824 [*M* − H]^-^), peak 18 (Fig. S18, *m/z* 578.27461 [*M* − H]^-^), and peak 19 (Fig. S19, *m/z* 547.36888 [*M* − H]^-^). The MS-MS spectra of peaks 14, 15, 16, 17, 18 and 19 revealed fragment ions at *m/z* 301.20403; *m/z* 161.02552; *m/z* 489.36367; *m/z* 191, 377, 403; *m/z* 577.27461, and *m/z* 369 and 443, respectively. These data are similar to published data of trihydroxy-octadecadienoic acid, 15-hydroxyhexadecanoic acid, 3α,24*R*,25-trihydroxytirucall-8-en-21-oic acid, 6α-hydroxyforsythide dimethyl ester, atractyloside G 2-*O*-β-d-glucopyranoside, and sibiricose A6, respectively [[43], [44], [45], [46], [47], [48]]. In addition, peaks 20 (Fig. S20, *m/z* 793.44611 [*M* − H]^-^), 21 (Fig. S21, *m/z* 505.35860 [*M* − H]^-^), 22 (Fig. S22, *m/z* 505.30604 [*M* − H]^-^), 23 (Fig. S23, *m/z* 563.36489 [*M* − H]^-^), and 24 (Fig. S24, *m/z* 489.36328 [*M* − H]^-^) were identified as zingibroside R1 (C_42_H_66_O_14_), quercetin 3-(2ʹʹ-acetylgalactoside) (C_23_H_22_O_13_), tinospinoside B and tinospinoside C (C_27_H_35_O_12_), apigenin-6-*C*-glu-8-*C*-ara (C_26_H_28_O_14_) and 17-hydroxy-17-methyl-4-estren-3-one 17-*O*-β-d-glucopyranoside (C_25_H_38_O_7_), respectively [18,44,[49], [50], [51]]. The MS-MS spectra of peaks 20, 22, 23 and 24 showed fragment ions at *m/z* 375 and 567; *m/z* 293, 471, and 489; *m/z* 293, 471, and 519; and *m/z* 325, 431, and 469, respectively. Finally, these compounds' identification was effected by comparison of the spectral data with literature data. The UPLC-QTOF-MS profiles showed that different plant parts of *Breonadia salicina* collected in different seasons produced both similar and dissimilar phytochemicals. This is the first report of these phytochemicals extracted from *B. salicina* plant parts. Furthermore, this is the first metabolomics evidence of the chemical variability of the different *B. salicina* plant parts collected during four successive seasons.

**Table 3:** The compounds identified by UPLC-QTOF-MS in the seasonal crude *B*. *salicina* extracts.

Peak No.	Rt (min)	Theoretical Mass [*M* − H]^-^(*m/z*)	Observed Mass [*M* − H]^-^(*m/z*)	Molecular formula	MS/MS fragment ions (*m/z*)	Compound Name	Compound class	Autumn extracts	Winter extracts	Spring extracts	Summer extracts	References
1	0.702	533.1738	533.17426	C_19_H_34_O_17_	191.05626	Quinic acid diglucoside	Quinic acid + monosaccharide	Stem, root	Stem, root	Stem, root, MeOH leaf	Stem, root	[32]
2	5.351	353.08685	353.08943	C_16_H_18_O_9_	191.05672	Chlorogenic acid	Quinic acids	Stem, root, leaf (MeOH & DCM)	Stem, root	Stem, root, leaf (MeOH & DCM)	Stem, root, leaf (MeOH & DCM)	[33]
3	5.204	390.388	390.11424	C_20_H_22_O_8_	389.11129	*trans*-Resveratroloside	Monomers	Stem	Stem	Stem	Stem	[34]
4	6.416	403.1319	403.12695	C_17_H_24_O_11_	404.13018	Oleoside 11-methylester	Tyrosols	Stem, root	Stem, root	Stem, root	Stem, root	[35]
5	7.210	515.1189	515.12236	C_25_H_24_O_12_	353,191	Dicaffeoyl quinic acid isomer: 3,4−diCQA	Quinic acids	Stem, root	Stem, root	Stem, root	Stem, root	[36]
6	7.723	537.1529	537.16508	C_32_H_26_O_8_	–	4,8,4′,8′-Tetramethoxy-[1,1′- biphenanthrene]-2,7,2′,7′- tetrol	Biphenanthrene derivatives	Stem, root	Stem, root	Stem, root	Stem, root	[37]
7	12.670	439	439.32578	C_29_H_44_O_3_	–	Pfaffic acid	Nortriterpene	Stem	Stem	Stem	Stem	[38]
8	0.746	369.0833	369.08500	C_16_H_18_O_10_	161,353	Ferulic acid 4-*O*-glucuronide	Phenolic glycosides	Stem, root	Stem, root	Root	Root	[35]
9	8.735	455.35412	455.31978	C_30_H_48_O_3_	–	Ursolic acid	Triterpenoid	Root, leaf (MeOH & DCM)	Root, leaf (MeOH & DCM)	Root, leaf (MeOH & DCM)	Root, leaf (MeOH & DCM)	[39]
10	0.760	191.1	191.05725	C_7_H_12_O_6_	–	Quinic acid	Quinic acids and derivatives	MeOH & DCM leaf	MeOH & DCM leaf	DCM leaf	MeOH & DCM leaf	[40]
11	5.218	389.1088	389.11215	C_16_H_22_O_11_	390.11531	Deacetyl asperuloside acid	Monoterpenoid	Stem, leaf (MeOH & DCM)	Stem, MeOH leaf	Stem, leaf (MeOH & DCM)	Stem, leaf (MeOH & DCM)	[41]
12	7.005	609.1464	609.15209	C_27_H_30_O_16_	463,447	Rutin	Flavonoid glycoside	MeOH & DCM leaf	MeOH leaf	MeOH & DCM leaf	MeOH & DCM leaf	[42]
13	7.290	515.5	515.12463	C_25_H_24_O_12_	353.09154	Di-*O*-caffeoylquinic acid	Quinic acid	MeOH & DCM leaf	MeOH & DCM leaf	MeOH & DCM leaf	MeOH leaf	[40]
14	16.805	327.2209	327.22003	C_18_H_32_O_5_	301.20403	Trihydroxy-octadecadienoic acid	Fatty acid	DCM leaf	DCM leaf	DCM leaf	DCM leaf	[43]
15	16.221	271.2351	271.23033	C_16_H_32_O_3_	161.02552	15-Hydroxyhexadecanoic acid	Fatty acid	DCM leaf	DCM leaf	DCM leaf	DCM leaf	[44]
16	16.652	490.3658	490.36367	C_30_H_50_O_5_	489.36367	3-α,24 R,25-Trihydroxytirucall-8-en-21-oic acid	Triterpenes	DCM leaf	DCM leaf	DCM leaf	DCM leaf	[45]
17	6.071	433.1424	433.13824	C_18_H_26_O_12_	191,377,403	6-α-Hydroxyforsythide dimethyl ester	Iridoid glucosides	Root	Root	Root	Root	[46]
18	14.230	578.2938	578.27461	C_27_H_46_O_13_	577.27461	Atractyloside G 2-*O*-β-d-glucopyranoside	Sesquiterpenoid	MeOH leaf	MeOH leaf	MeOH leaf	MeOH leaf	[47]
19	14.449	547.1631	547.36888	C_23_H_32_O_15_	369,443	Sibiricose A6	Oligosaccharide ester	DCM leaf	–	DCM leaf	DCM leaf	[48]
20	12.077	793.43650	793.44611	C_42_H_66_O_14_	375,567	Zingibroside R1	dammaranae-type triterpenoid saponin	Stem, root	Stem, root	Stem, root	Stem, root	[18]
21	14.807	505.0979	505.35860	C_23_H_22_O_13_	–	Quercetin 3-(2ʹʹ-acetylgalactoside)	Flavonoid	DCM leaf	DCM leaf	DCM leaf	DCM leaf	[44]
22	13.583	505.0	505.30604	C_27_H_35_O_12_	293,471,489	Tinospinoside B and Tinospinoside C	diterpene glycosides	DCM leaf	DCM leaf	DCM leaf	DCM leaf	[49]
23	13.856	563.0	563.36489	C_26_H_28_O_14_	293,471,519	Apigenin-6-*C*-glu-8-*C*-ara	Flavonoid	DCM leaf	DCM leaf	DCM leaf	DCM leaf	[50]
24	14.435	489.2249	489.36328	C_25_H_38_O_7_	325,431,469	17-Hydroxy-17-methyl-4- estrene-3-one 17-*O*-β-d-glucopyranoside	Glycoside	DCM leaf	DCM leaf	DCM leaf	DCM leaf	[51]

### 3.2 Quality assurance by means of UPLC-MS

#### 3.2.1 Untargeted UPLC-MS analysis

**Fig. 2:**
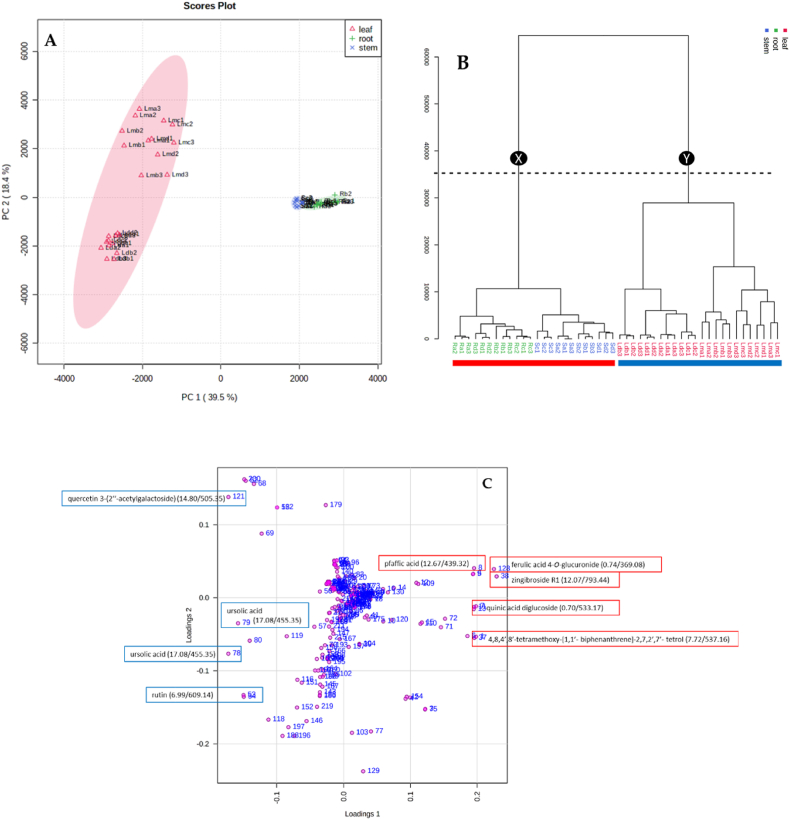
(**A**) Principal component analysis (PCA) of clusters of leaf, stem bark and root crude extracts collected in autumn, winter, spring, and summer. (**B**) Dendrogram acquired by HCA of the LC-MS data obtained from the leaf, stem bark and root samples from four different seasons (n = 48). Branch X (red) represents root and stem bark samples; branch Y (blue) is formed by methanol and dichloromethane leaf extracts. (**C**) Loadings score plot obtained from constituents shown by red (root) and red (stem bark) rectangles contributing to the clustering of Branch X (red), while compounds in blue (leaf) rectangles contribute to the Branch Y (blue) clusters. The constituent specified by a red rectangle (ferulic acid 4-*O*-glucuronide: 0.74/369.08) is the first variable indicated by the VIP plot.

**Fig. 3:**
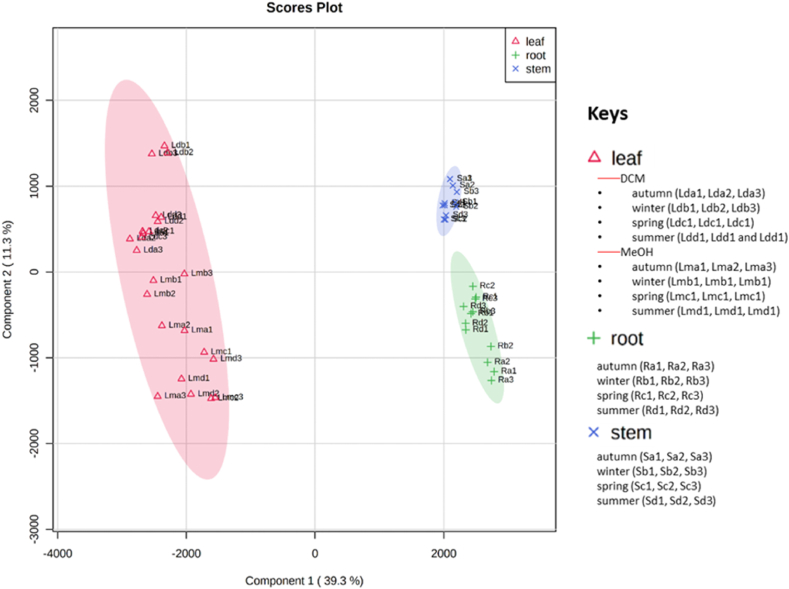
PLS-DA of clusters of the dendrogram.

**Fig. 4:**
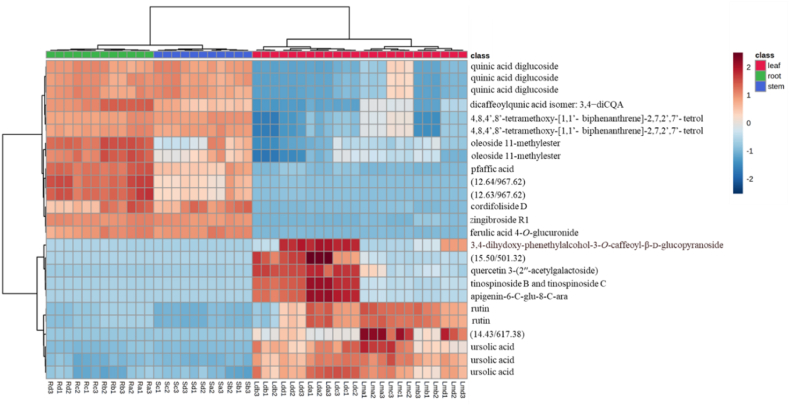
Heatmap of 25 peaks produced by stem, root and leaf extracts of *B. salicina* (n = 48).

The PCA model was created from the UPLC-QTOF-MS fingerprints of the leaf, stem bark and root samples of the four different seasons, to determine any trends. A scores plot was established using the first principal component (39.5 %) and second principal component (18.4 %), together accounting for 57.9 % of the variation in the data. The scores plot was coloured based on the three different parts of the plant (leaf, stem bark and root) samples as shown in Fig. 2A. Generally, two key clusters that are associated with the different parts of the plant (leaf, stem bark and root) samples are observed. The group encompassing the leaves was separated along negative PC1 (cluster 1) while the stem and the root samples were clustered along positive PC1 (cluster 2). In cluster 1, there was a clear separation between the dichloromethane and methanol leaf extracts along PC2. The PLS-DA analysis (Fig. 3) enabled identification of the compounds contributing to the different dendrogram clusters. A scores plot was constructed using the first (39.3 %) and second (11.3 %) components, contributing 50.6 % of the variation (as shown in Fig. 3). Branch red (X), which consists of the stem bark and root samples was separated along negative PC1; and branch blue (Y), which consists of the methanol leaf and dichloromethane leaf samples was distributed on the positive PC1. Furthermore, there was a clear separation of the dichloromethane and methanol leaf extracts along PC2. In addition, separation of the stem bark and root samples can be observed along PC1. There was also a clear separation of the root samples; the autumn samples (Ra1, Ra2, Ra3) were further separated from the other seasons, indicating that the chemistry of the autumn root samples differs from that of the other seasons (as shown in Fig. 3). The evaluation of the PLS-DA models revealed clear separations and grouping from the datasets. The separations observed give insight into the chemical variability and correlation between the three different plant parts in each season. A hierarchical cluster analysis (HCA) dendrogram (Fig. 2B) was also constructed to further explore the chemical variability within the samples. Branch X (red) consists of stem and root samples while branch Y consist of leaf samples. Under branch X, the stem and root samples are further separated indicating distinct chemistry that is not visible in the PCA plot. The sequence of clustering of the stem bark extracts is as follows: summer (Sd1, Sd2, Sd3), winter (Sb1, Sb2, Sb3), autumn (Sa1, Sa2, Sa3) and spring (Sc1, Sc2, Sc3). However, the root samples reveal a different sequence as follows: spring (Rc1, Rc2, Rc3), winter (Rb1, Rb2, Rb3), summer (Rd1, Rd2, Rd3) and autumn (Ra1, Ra2, Ra3). In branch Y (blue) the sequence of the clustering of dichloromethane extracts is different from that of the methanol extracts. The constituents causing most of the discrimination were found using the Variable importance for project (VIP) scores (Fig. 2C). In the leaf samples, ursolic acid (*m/z* 455.35/Rt 17.08 min), rutin (*m/z* 609.14/Rt 6.99 min) and quercetin 3-(2ʹʹ-acetylgalactoside) (*m/z* 505.35/Rt 14.80 min) were identified as markers while zingibroside R1 (*m/z* 793.44/Rt 12.07 min), quinic acid diglucoside (*m/z* 533.17/Rt 0.70 min), pfaffic acid (*m/z* 439.32/Rt 12.67 min), 4,8,4′,8′-tetramethoxy-[1,1′-biphenanthrene]-2,7,2′,7′-tetrol (*m/z* 537.15/Rt 7.72 min) and *m/z* 967.62 (Rt 12.64 min) were identified as markers for the root and 4,8,4′,8′-tetramethoxy-[1,1′-biphenanthrene]-2,7,2′,7′-tetrol (*m/z* 537.15/Rt 7.72 min), ferulic acid 4-*O*-glucuronide (*m/z* 369.08/Rt 0.74 min) and quinic acid diglucoside *m/z* 533.17/Rt 0.70 min) markers for stem bark. In the heatmap (Fig. 4), the concentrations of rutin, ursolic and unknown compound (*m/z* 617.38/Rt 14.43 min) were higher in the leaf samples compared to the root and stem bark samples. On the other hand, the concentrations of ferulic acid 4-*O*-glucuronide, oleoside 11-methylester, pfaffic acid, 4,8,4′,8′-tetramethoxy-[1,1′-biphenanthrene]-2,7,2′,7′-tetrol, cordifoliside D, zingibroside R1, dicaffeoylquinic acid isomer: 3,4−diCQA, quinic acid diglucoside and two unknown compounds (*m/z* 967.62, Rt 12.63; *m/z* 967.62, Rt 12.64 min) were higher in the root and stem bark samples compared to the leaf samples. In the dichloromethane and methanol leaf extracts, ursolic acid was moderate for all seasons while rutin was not found in the dichloromethane leaf samples collected in winter.

**Fig. 5:**
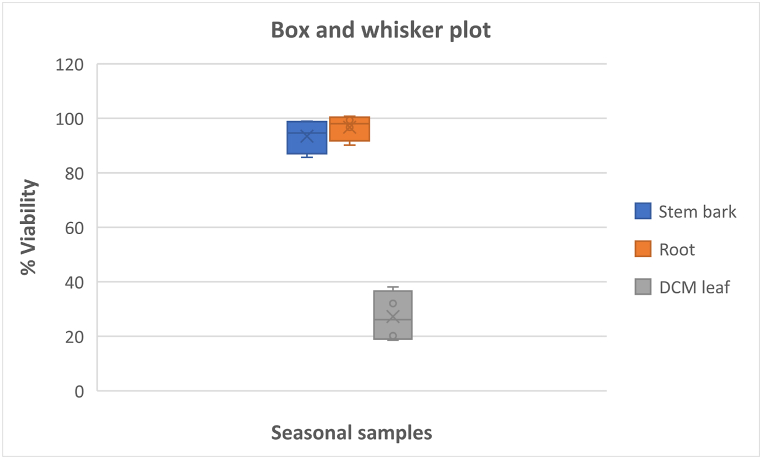
A box-and-whiskers plot of differentially expressed metabolites of the samples in the antimalarial tests.

**Fig. 6:**
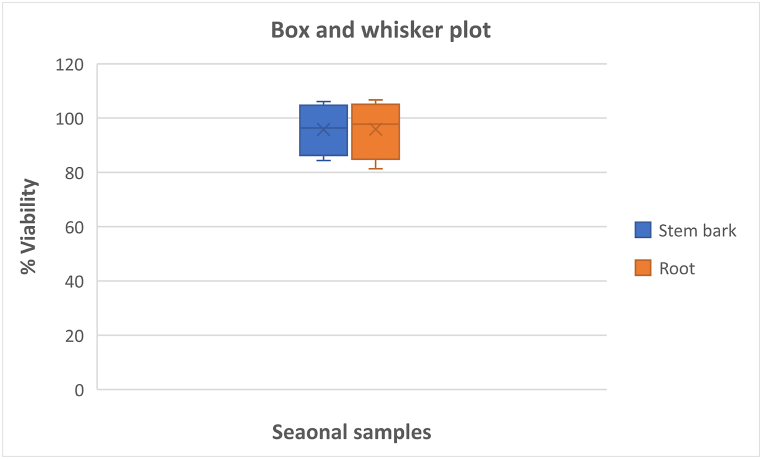
A box-and-whiskers plot of differentially expressed metabolites of the samples in the antitrypanosomal tests.

### 3.3 Pharmacological activities of the seasonal extracts

#### 3.3.1 Antimalarial activity

The chemical variability of the seasonal plant samples (autumn, winter, spring and summer) was evaluated against *Plasmodium falciparum* (3D7 strain) parasites. The results indicate that the leaf extracts had the highest antimalarial activities compared to the root and stem bark extracts (Table 4). Furthermore, this study shows that the dichloromethane leaf extracts collected in each season had higher antimalarial activities than the methanol leaf extracts. This can be attributed to the fact that the dichloromethane leaf extracts in each season, as revealed by UPLC-QTOF-MS, produced compounds which were different from the compounds found in the methanol leaf, stem bark and root extracts. The chemical profiles of the dichloromethane leaf extracts show the presence of metabolites including trihydroxy-octadecadienoic acid, 15-hydroxyhexadecanoic acid, 3α,24 R, 25-trihydroxytirucall-8-en-21-oic acid, quercetin 3-(2ʹʹ-acetylgalactoside), tinospinoside B, tinospinoside C, apigenin-6-*C*-glu-8-*C*-ara and 17-hydroxy-17-methyl-4-estren-3-one 17-*O*-β-d-glucopyranoside (as shown in Table 3). However, they were not found in the methanol leaf, root, and stem bark extracts (as presented in Table 3). Therefore, these metabolites might have contributed to the strong antimalarial activities of the dichloromethane leaf extracts of *Breonadia salicina* in each season. In addition, the highest antimalarial activity of the dichloromethane leaf extracts was found in autumn, followed by summer, winter and spring, with viabilities of 18.57 ± 1.99 %, 20.21 ± 5.19 %, 32.07 ± 4.91 % and 38.11 ± 5.07 % at 50 μg/mL, respectively (Table 4); and IC_50_ values of 7.903 ± 0.060 μg/mL, 15.26 ± 0.059 μg/mL, 18.15 ± 0.074 μg/mL and 19.40 ± 0.065 μg/mL at 300 μg/mL, respectively (Table 4). The dichloromethane leaf extracts had different pharmacological activities, even though the UPLC-QTOF-MS chromatograms showed that the dichloromethane leaf extracts in each season produced the same metabolites. However, the compounds produced in autumn had the highest intensity compared to the compounds found in other seasons. However, the methanol leaf extracts in autumn, winter, summer and spring did not significantly decrease the viability of *Plasmodium falciparum* (82.13 ± 7.28 %, 78.07 ± 1.04 %, 77.66 ± 1.25 % and 53.44 ± 6.22 %, respectively) at 50 μg/mL (Table 4) compared to the dichloromethane leaf extracts; whereas the reference drug chloroquine had an IC_50_ value of 0.03 μg/mL. Moreover, the same trend observed in the biological assays corresponds to the chemistry of the methanol leaf extracts and the dichloromethane leaf extracts. Chemometric analysis of the data indicates that the chemistry of the stem and root is similar and different from the chemistry of the leaf (as shown in Fig. 2A). Furthermore, chemometrics analysis shows a clear separation of the dichloromethane and methanol leaf extracts along PC2 (as shown in Fig. 2A), indicating that the chemistry and bioactivity of the methanol and dichloromethane leaf extracts in each season differ. The markers identified by the VIP plot (Fig. 2C) and heatmap (Fig. 4) indicate that apigenin-6-*C*-glu-8-*C*-ara, tinospinoside B, tinospinoside C, quercetin 3-(2ʹʹ-acetylgalactoside), and 3,4-dihydoxyphenethylalcohol-3-*O*-caffeoyl-β-d-glucopyranoside found in the dichloromethane leaf samples did not occur in the methanol leaf, stem bark and root samples. Therefore, variations in the concentration of these metabolites affected the antimalarial activities of the dichloromethane leaf extracts against *Plasmodium falciparum* in each season. A PCA model was created to assess the relationship between the presence of certain compounds and the extract bioactivity. A scores plot was established using the first and second components, accounting for 57.9 % of the variation in the data (Fig. 2A). Fig. 2A shows that the active samples (Red) are distributed along PC2 and the non-active samples (Green and Blue) are clustered along PC1. Compounds showing significant contribution to the separation were found using a VIP plot (Fig. 2C). This is the first study showing that the presence of certain phytochemicals contributes to the antimalarial activities of *B. salicina* in autumn, winter, spring and summer.

**Table 4:** Antimalarial activities of the methanolic and dichloromethane extracts of plant materials harvested in different seasons.

Samples	pLDH (% viability ± SD) 50 μg/mL	IC_50_ (μg/mL) 300 μg/mL
Stem bark extract (autumn)	90.95 ± 8.10^a^	–
Stem bark extract (winter)	98.94 ± 0.80^a^	–
Stem bark extract (spring)	85.69 ± 1.51^a^	–
Stem bark extract (summer)	98.19 ± 2.88^a^	–
Root extract (autumn)	90.14 ± 8.51^b^	–
Root extract (winter)	100.75 ± 1.84^b^	–
Root extract (spring)	99.33 ± 3.56^b^	–
Root extract (summer)	96.69 ± 6.28^b^	–
DCM leaf extract (autumn)	18.57 ± 1.99^a,b,c^	7.903 ± 0.06^a^
DCM leaf extract (winter)	32.07 ± 4.91^a,b,c^	18.15 ± 0.07^a,b^
DCM leaf extract (spring)	38.11 ± 5.07^a,b,c^	19.40 ± 0.06^a,b,c^
DCM leaf extract (summer)	20.21 ± 5.19^a,b,c^	15.26 ± 0.05^a,b,c^
MeOH leaf extract (autumn)	82.13 ± 7.28^c^	–
MeOH leaf extract (winter)	78.07 ± 1.04^c^	–
MeOH leaf extract (spring)	53.44 ± 6.22^c^	–
MeOH leaf extract (summer)	77.66 ± 1.25^c^	–

Notes: Different superscripts mean significant differences in one-way ANOVA at *p* < 0.05. Data are reported as mean ± SD (n = 3). In the % parasite viability test: ^a^—The stem bark samples in each season differed significantly to the dichloromethane leaf samples in each season; ^b^—Root samples in each season differed significantly to the dichloromethane leaf samples in each season; and ^c^—The dichloromethane leaf samples in each season differed significantly to the methanol leaf samples in each season. In the IC_50_ test: ^a^—The dichloromethane leaf samples in autumn differed significantly to the dichloromethane leaf samples in (winter, spring and summer); ^b^—Dichloromethane leaf extracts in winter differed significantly from the dichloromethane leaf extracts in spring and summer; and ^c^—The dichloromethane leaf extracts in spring differed significantly from the dichloromethane leaf extracts in summer. Some differentially expressed metabolites were summarised using a box-and-whiskers plots (Fig. 5).

#### 3.3.2 Antitrypanosomal activity

The chemical variability of fresh seasonal samples (autumn, winter, spring, and summer) was evaluated against *Trypanosoma brucei* (strain 427) parasites. The results show that the leaf extracts had the highest antitrypanosomal activity (Table 5). Furthermore, UPLC-QTOF-MS reveals that the methanol leaf extracts contained metabolites such as atractyloside G 2-*O*-β-d-glucopyranoside, di-*O*-caffeoylquinic acid, deacetyl asperuloside acid, rutin and quinic acid (as shown in Table 3). However, these phytochemicals were not found in the other extracts (Table 3). Therefore, these phytochemicals might have been responsible for the antitrypanosomal activities of the dichloromethane and methanol leaf extracts of *Breonadia salicina* in each season. In addition, both the methanol and dichloromethane leaf extracts from all four seasons display the highest antitrypanosomal activity, except for the methanol leaf extracts in winter. The highest antitrypanosomal activity of the methanol leaf extracts was found in autumn, followed by summer, spring and winter, with viabilities of 5.84 ± 0.38 %, 9.05 ± 0.80 %, 26.66 ± 3.91 % and 85.56 ± 3.52 %, respectively, at 50 μg/mL; and IC_50_ values of 12.0 ± 0.36 μg/mL, 10.6 ± 0.07 μg/mL, 5.2 ± 0.74 μg/mL at 300 μg/mL, respectively (Table 5); the winter methanol leaf extract did not show any activity. Furthermore, the highest antitrypanosomal activity of the dichloromethane leaf extracts was found in autumn, followed by summer, winter and spring, with viabilities of 3.50 ± 0.59 %, 3.85 ± 0.10 %, 4.13 ± 0.06 % and 29.47 ± 1.25 %, respectively, at 50 μg/mL (Table 5); with IC_50_ values of 4.6 ± 1.82 μg/mL, 4.0 ± 0.08 μg/mL, 5.1 ± 0.30 μg/mL and 5.1 ± 0.72 μg/mL at 300 μg/mL, respectively (Table 5). The dichloromethane leaf extracts had different biological activities; this was because the UPLC-QTOF-MS chromatographs showed that the dichloromethane leaf extracts in each season produced the same metabolites. However, the metabolites produced in autumn had the highest intensity compared to the compounds found in other seasons. Chemometrics analysis of the data shows a clear separation of the dichloromethane and methanol leaf extracts along PC2 (as shown in Fig. 2A). Seasonal variations are observed in each plant part in which autumn and winter samples were closely clustered, compared to the spring and summer in the methanol leaf plant parts. The markers identified by the VIP plot (Fig. 2C) and heatmap (Fig. 4) indicate ursolic acid and rutin in the methanol leaf samples but not in the stem bark and root samples. Furthermore, apigenin-6-*C*-glu-8-*C*-ara, tinospinoside B, tinospinoside C, quercetin 3-(2ʹʹ-acetylgalactoside), and 3,4-dihydoxyphenethylalcohol-3-*O*-caffeoyl-β-d-glucopyranoside were found in the dichloromethane leaf samples but not in the methanol leaf, stem bark and root samples. Therefore, we suggest that these metabolites contribute to the variation in antitrypanosomal activities of the methanol and dichloromethane leaf extracts against *Trypanosoma brucei.* The reference drug pentamidine was used as positive control for all the tested crude extracts in autumn, winter, spring and summer; and had an IC_50_ value of 10.2 ± 0.07 μg/mL. To the best of our knowledge, this is the first study evaluating compounds that might be responsible for the antitrypanosomal activities of *Breonadia salicina.*

**Table 5:** Antitrypanosomal activities of the seasonal extracts.

Samples	*T. brucei* (% viability ± SD) 50 μg/mL	IC_50_ (μg/mL) 300 μg/mL
Stem bark extract (autumn)	100.62 ± 0.26^a,b^	–
Stem bark extract (winter)	92.04 ± 3.82^a,b^	–
Stem bark extract (spring)	106.07 ± 7.70^a,b^	–
Stem bark extract (summer)	84.32 ± 8.56^a,b^	–
Root extract (autumn)	100.15 ± 1.19^a,b,c^	–
Root extract (winter)	95.37 ± 6.52^a,b,c^	–
Root extract (spring)	106.65 ± 8.84^a,b,c^	–
Root extract (summer)	81.32 ± 4.78^a,b,c^	–
DCM leaf extract (autumn)	4.13 ± 0.06^b,c^	4.6 ± 1.82^a^
DCM leaf extract (winter)	3.50 ± 0.59^b,c^	5.1 ± 0.30^a,b,c^
DCM leaf extract (spring)	29.47 ± 1.25^b,c^	5.1 ± 0.72^a,b,d,e^
DCM leaf extract (summer)	3.85 ± 0.10^b,c^	4.0 ± 0.08^a,b,d,f^
MeOH leaf extract (autumn)	5.84 ± 0.38^b,c^	12.0 ± 0.36^a,c,e,f^
MeOH leaf extract (winter)	85.56 ± 3.52^b,c^	–
MeOH leaf extract (spring)	26.66 ± 3.91^b,c^	5.2 ± 0.74^a,b,d^
MeOH leaf extract (summer)	9.05 ± 0.80^b,c^	10.6 ± 0.07^a,c,e,f^

Notes: Different superscripts mean significant differences in one-way ANOVA at *p* < 0.05. Data are reported as mean ± SD (n = 3). In the parasite viability test: ^a^—The stem bark samples in each season did not differ significantly from the root samples in each season; ^b^—Stem bark samples in each season differed significantly from the dichloromethane and methanol leaf samples in each season; and ^c^—The root samples in each season differed significantly from the dichloromethane and methanol leaf samples in each season. The IC_50_ determination: ^a^—The dichloromethane leaf samples in autumn did not differ significantly from the dichloromethane leaf samples in winter, spring and summer, and the methanol leaf samples in autumn, spring and summer; ^b^—The dichloromethane leaf samples in winter did not differ significantly from the dichloromethane leaf samples in spring and summer, and the methanol leaf samples in spring; ^c^—Dichloromethane leaf samples in winter did not differ significantly from the methanol leaf samples in autumn and summer; ^d^—The dichloromethane leaf samples in spring did not differ significantly from the dichloromethane leaf samples in summer and methanol leaf samples in summer; ^e^—Dichloromethane leaf samples in spring differed significantly from the methanol leaf samples in autumn and summer; and ^f^—The dichloromethane leaf samples in summer differed significantly from the methanol leaf samples in autumn and summer. Some differentially expressed metabolites are summarised using a box-and-whiskers plot (shown in Fig. 6).

### 3.4 Biochemometric analysis

**Fig. 7:**
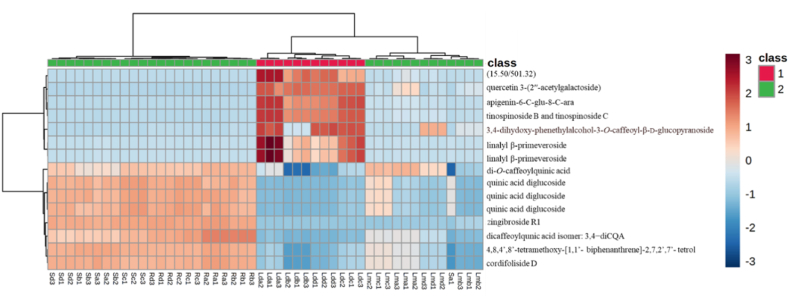
Heatmap of 15 peaks in 16 samples of *B. salicina* from the dichloromethane leaf extracts (Class 1) and Class 2 (methanol leaf, stem bark and root extracts).

**Fig. 8:**
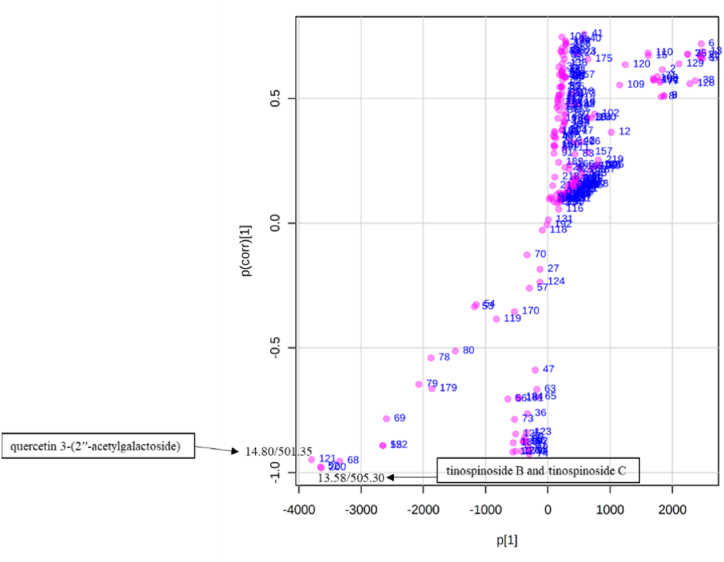
Loadings plot showing the biomarkers (Rt/molecular ion *m/z*) from the dichloromethane leaf extracts (Class 1) and Class 2 (methanol leaf, stem bark and root extracts).

**Fig. 9:**
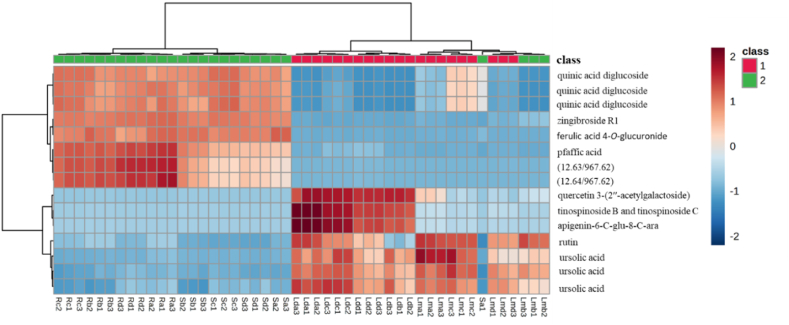
Heatmap of 15 peaks in 16 samples of *B. salicina* from the dichloromethane and methanol leaf extracts (Class 1) and Class 2 (winter methanol leaf, stem bark and root extracts).

**Fig. 10:**
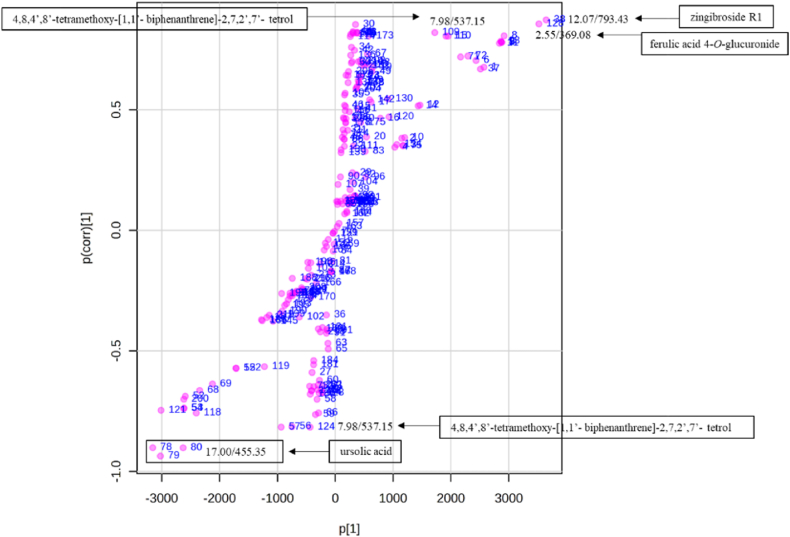
Loadings plot showing the biomarkers (Rt/molecular ion *m/z*) of the dichloromethane and methanol leaf extracts (Class 1) and Class 2 (winter methanol leaf, stem bark and root extracts).

To determine the link between the variation in chemotype, antimalarial, and antitrypanosomal activities, a biochemometric analysis was performed. Samples were classified into two groups according to their antimalarial and antitrypanosomal activities. The dichloromethane leaf extracts had the highest antimalarial activities in each season and were assigned to Class 1, while the methanol leaf, stem, and root extracts were assigned to Class 2 (Fig. 7). The link between the identified metabolites and bioactivity was determined by the constructed OPLS-DA model. The selection of compounds contributing significantly to separation of the two classes was carried out using a VIP plot (Fig. 8). The loadings plot shows that quercetin 3-(2ʹʹ-acetylgalactoside) (Rt 14.80 min), tinospinoside B and tinospinoside C (Rt 13.58 min) are the most significant biomarkers for Class 1 (as shown in Fig. 8). This is the first report identifying any biomarker for *B. salicina.* The biomarker concentrations in all samples were visualised by means of a heatmap of the predominant phytochemicals (Fig. 7). The concentrations of linalyl β-primeveroside were higher in Class 1. On the other hand, the concentrations of 3,4-dihydoxyphenethylalcohol-3-*O*-caffeoyl-β-d-glucopyranoside, tinospinoside B, tinospinoside C, apigenin-6-*C*-glu-8-*C*-ara, and quercetin 3-(2ʹʹ-acetylgalactoside) were moderate in Class 1 (as presented in Fig. 7). In Class 2, the concentrations of di-*O*-caffeoylquinic acid, quinic acid diglucoside, zingibroside R1, dicaffeoylquinic acid isomer: 3,4−diCQA, 4,8,4′,8′-tetramethoxy-[1,1′-biphenanthrene]-2,7,2′,7′-tetrol and cordifoliside D were moderate (Fig. 7). The variation in the antimalarial activities of the dichloromethane leaf extracts (Class 1) in each season is associated with the concentrations of linalyl β-primeveroside, 3,4-dihydoxyphenethylalcohol-3-*O*-caffeoyl-β-d-glucopyranoside, tinospinoside B, tinospinoside C, apigenin-6-*C*-glu-8-*C*-ara, and quercetin 3-(2ʹʹ-acetylgalactoside). Furthermore, the biochemometric analysis of the antitrypanosomal activity of the active dichloromethane and methanol leaf extracts was determined. The dichloromethane and methanol leaf extracts were assigned as Class 1 since they had the highest antitrypanosomal activities in each season, except for the methanol leaf extracts in winter (as shown in Fig. 9). However, the stem bark and root extracts, and the winter methanol leaf extract, which were assigned to Class 2, showed the lowest antitrypanosomal activities (Fig. 9). The markers identified by the VIP plot show that ursolic acid (Rt 17.00 min), 4,8,4′,8′-tetramethoxy-[1,1′-biphenanthrene]-2,7,2′,7′-tetrol (Rt 7.98 min), ferulic acid 4-*O*-glucuronide (Rt 2.55 min), and zingibroside R1 (Rt 12.07 min) are the most important for Class 1 and Class 2 (as shown in Fig. 10). In the heatmap (Fig. 9), the concentrations of ursolic acid, rutin and quercetin 3-(2ʹʹ-acetylgalactoside) were moderate in Class 1. However, apigenin-6-*C*-glu-8-*C*-ara, tinospinoside B and tinospinoside C were higher in Class 1 (as shown in Fig. 9). In addition, the concentrations of quinic acid diglucoside, zingibroside R1, ferulic acid 4-*O*-glucuronide, pfaffic acid and unknown compounds (*m/z* 967.62./Rt 12.63 min and *m/z* 967.62/Rt 12.64 min) were moderate in Class 2 (Fig. 9). No specific compounds can be identified as being responsible for the antimalarial and antitrypanosomal activities of the dichloromethane and methanol leaf extracts, highlighting the complicated interactions between the secondary metabolites present. Therefore, we conclude that the major compounds of *B. salicina* contribute to the chemical, antimalarial and antitrypanosomal variations among the dichloromethane and methanol leaf extracts.

## 4 Conclusions

In this study we set out to determine the phytochemical composition, antimalarial and antitrypanosomal activities of *Breonadia salicina* plant parts. Twenty-four compounds were identified tentatively, using mass spectral data from seasonal samples. We have identified these metabolites for the first time in *B. salicina*. Furthermore, we have shown that the dichloromethane leaf extracts in each season had the highest antimalarial activity compared to the methanol leaf, stem bark and root extracts. In addition, the methanol and dichloromethane leaf extracts in autumn, winter, spring and summer displayed the highest antitrypanosomal activity, except for the methanol leaf extracts in winter. The chemical profiles revealed that different plant parts of *Breonadia salicina* produce similar and dissimilar metabolites. Therefore, we conclude that continuous variations could occur with variations in distribution of metabolites in plant parts. Chemometrics analysis of the data obtained indicates that the chemistry of the stem and root are similar and different from the chemistry of the leaf. Further studies are required that include a greater range of populations, including samples from different locations in Vhembe region in Limpopo Province or spread throughout South Africa. This would allow a comprehensive comparison to be made of the chemical variation within the stem bark, roots and leaves of *Breonadia salicina.*

## Funding statement

This work was financially supported by the 10.13039/501100001321National Research Foundation, 10.13039/501100004290Sasol Foundation, and the 10.13039/501100008976University of Venda RPC (Research and Publications Committee). The parasite screening was funded by the Grand Challenges Africa programme (GCA/DD/rnd3/032).

## Data availability statement

Data will be made available on request.

## CRediT authorship contribution statement

**Dorcas Tlhapi:** Writing - review & editing, Writing - original draft, Visualization, Validation, Methodology, Investigation, Data curation, Conceptualization. **Isaiah Ramaite:** Writing - review & editing, Supervision, Conceptualization. **Chinedu Anokwuru:** Writing - review & editing, Supervision, Conceptualization. **Teunis van Ree:** Writing - review & editing, Supervision, Conceptualization. **Ntakadzeni Madala:** Writing - review & editing. **Heinrich Hoppe:** Writing - review & editing, Methodology.

## Declaration of competing interest

The authors declare that they have no known competing financial interests or personal relationships that could have appeared to influence the work reported in this paper.

## References

[1] Soni U., Brar S., Gauttam V.K. (2015). Effect of seasonal variation on secondary metabolites of medicinal plants. Int. J. Pharma Sci. Res..

[2] Sultana R., Majid N., Nissar S., Rather A.M. (2018). Seasonal variation of phytochemicals. Int. J. Pharm. Biol. Sci..

[3] Demuner A.J., Almeida Barbosa L.C., Gonçalves Magalhaes C., Da Silva C.J., Alvares Maltha C.R., Lelis Pinheiro A. (2011). Seasonal variation in the chemical composition and antimicrobial activity of volatile oils of three species of *Leptospermum* (Myrtaceae) grown in Brazil. Molecules.

[4] Ncube B., Finnie J.F., Van Staden J. (2011). Seasonal variation in antimicrobial and phytochemical properties of frequently used medicinal bulbous plants from South Africa. S. Afr. J. Bot..

[5] Lubbe A., Gude H., Verpoorte R., Choi Y.H. (2013). Seasonal accumulation of major alkaloids in organs of pharmaceutical crop Narcissus Carlton. Phytochemistry (Elsevier).

[6] Tomar N.S., Sharma M., Agarwal R.M. (2015). Phytochemical analysis of *Jatropha curcas* L. during different seasons and developmental stages and seedling growth of wheat (*Triticum aestivum* L) as affected by extracts/leachates of *Jatropha curcas* L. Physiol. Mol. Biol. Plants.

[7] Gololo S.S., Shai L.J., Agyei N.M., Mogale M.A. (2016). Effect of seasonal changes on the quantity of phytochemicals in the leaves of three medicinal plants from Limpopo province. South Africa. J. Pharmacogn. Phytotherapy.

[8] Nndwammbi M., Ligavha-Mbelengwa M.H., Anokwuru C.P., Ramaite I.D.I. (2018). The effects of seasonal debarking on physical structure, polyphenolic content and antibacterial and antioxidant activities of *Sclerocarya birrea* in the Nylsvley nature reserve. S. Afr. J. Bot..

[9] Bhardwaj S., Rashmi V., Parcha V. (2019). Effect of seasonal variation on chemical composition and physicochemical properties of *Hedychium spicatum* rhizomes essential oil. J. Essent. Oil Bear. Pl..

[10] Kibungu W.C., Fri J., Clarke A.M., Otigbu A., Akum Njom H. (2021). Seasonal variation in antimicrobial activity of crude extracts of Psammaplysilla sp. 1 from Phillips Reef. S. Afri. Internet J. Microbiol..

[11] Ahmad I., Ahmad M.S.A., Ashraf M., Hussain M., Ashraf M.Y. (2011). Seasonal variation in some medicinal and biochemical ingredients in *Mentha longifolia* (L.) Huds. Pakistan J. Bot..

[12] Szakiel A., Pączkowski C., Henry M. (2011). Influence of environmental abiotic factors on the content of saponins in plants. Phytochemistry Rev..

[13] Dhami N., Mishra A.D. (2015). Phytochemical variation: how to resolve the quality controversies of herbal medicinal products?. J. Herb. Med..

[14] Falasca A., Melck D., Paris D., Saviano G., Motta A., Iorizzi M. (2014). Seasonal changes in the metabolic fingerprint of *Juniperus communis* L. berry extracts by ^1^H-NMR-based metabolomics. Metabolomics.

[15] Farag M.A., Gad H.A., Heiss A.G., Wessjohann L.A. (2014). Metabolomics driven analysis of six Nigella species seeds via UPLC-qTOF-MS and GC–MS coupled to chemometrics. Food Chem..

[16] Heyman H.M., Senejoux F., Seibert I., Klimkait T., Maharaj V.J., Meyer J.J.M. (2015). Identification of anti-HIV active dicaffeoylquinic-and tricaffeoylquinic acids in *Helichrysum populifolium* by NMR-based metabolomic guided fractionation. Fitoterapia.

[17] Gazim Z.C., Amorim A.C.L., Hovell A.M.C., Rezende C.M., Nascimento I.A., Ferreira G.A., Dga, Cortez D.A.G. (2010). Seasonal variation, chemical composition, and analgesic and antimicrobial activities of the essential oil from leaves of *Tetradenia riparia* (Hochst.) Codd in Southern Brazil. Molecules.

[18] Fan Y., Li Y., Wu Y., Li L., Wang Y., Li Y. (2017). Identification of the chemical constituents in Simiao Wan and rat plasma after oral administration by GC-MS and LC-MS. Evid. Based Complementary Altern. Med..

[19] Kim N.K., Park H.M., Lee J., Ku K.M., Lee C.H. (2015). Seasonal variations of metabolome and tyrosinase inhibitory activity of *Lespedeza maximowiczii* during growth periods. J. Agric. Food Chem..

[20] Scognamiglio M., D'Abrosca B., Esposito A., Fiorentino A. (2015). Chemical composition and seasonality of aromatic mediterranean plant species by NMR-based metabolomics. J. Anal. Methods Chem..

[21] Sibandze G.F. (2009).

[22] Ali S., Umar A.Z., Asmau M., Deepa S., Milli J., Fatima H. (2019). In vitro antitrypanosomal activity of *Breonadia salicina* on *Trypanasoma brucei brucei*. Int. J. Pharma Sci. Res..

[23] A Salem M., Perez de Souza L., Serag A., Fernie A.R., Farag M.A., Ezzat S.M., Alseekh S. (2020). Metabolomics in the context of plant natural products research: from sample preparation to metabolite analysis. Metabolites.

[24] Ayo S.G., Habila J.D., Achika J.I., Akinwande O.O. (1930). Isolation and characterization of 2,4-dihydroxycinnamic acid from the stem bark of *Adina microcephala* Delile. Chem. Soc. Nig..

[25] Mahlo S.M., McGaw L.J., Eloff J.N. (2013). Antifungal activity and cytotoxicity of isolated compounds from leaves of *Breonadia salicina*. J. Ethnopharmacol..

[26] Nvau B.J., Sami B., Ajibade O.S., Gray I.A., Igoli J.O. (2019). Adicardin and other coumarins from *Breonadia salicina* (Vahl) Hepper. Trop. J. Nat. Prod. Res..

[27] Tlhapi D.B., Ramaite I.D., Anokwuru C.P. (2021). Metabolomic profiling and antioxidant activities of *Breonadia salicina* using ^1^H-NMR and UPLC-QTOF-MS analysis. Molecules.

[28] Martins D., Nunez C.V. (2015). Secondary metabolites from Rubiaceae species. Molecules.

[29] Lunga M.J., Chisango R.L., Weyers C., Isaacs M., Taylor D., Edkins A.L., Khanye S.D., Hoppe H.C., Veale C.G. (2018). Expanding the SAR of nontoxic antiplasmodial indolyl‐3‐ethanone ethers and thioethers. ChemMedChem.

[30] Scovill J., Blank E., Konnick M., Nenortas E., Shapiro T. (2002). Antitrypanosomal activities of *Tryptanthrins*. Antimicrob. Agents Chemother..

[31] Sandasi M., Kamatou G.P., Viljoen A.M. (2011). Chemotaxonomic evidence suggests that *Eriocephalus tenuifolius* is the source of Cape chamomile oil and not *Eriocephalus punctulatus*. Biochem. Systemat. Ecol..

[32] Granato D., do Prado-Silva L., Alvarenga V.O., Zielinski A.A., Bataglion G.A., de Morais D.R., Eberlin M.N., Sant'Ana A.D.S. (2016). Characterization of binary and ternary mixtures of green, white and black tea extracts by electrospray ionization mass spectrometry and modeling of their in vitro antibacterial activity. LWT--Food Sci. Technol..

[33] Peeters L., Van der Auwera A., Beirnaert C., Bijttebier S., Laukens K., Pieters L., Hermans N., Foubert K. (2020). Compound characterization and metabolic profile elucidation after in vitro gastrointestinal and hepatic biotransformation of an *Herniaria hirsuta* extract using unbiased dynamic metabolomic data analysis. Metabolites.

[34] Goufo P., Singh R.K., Cortez I. (2020). A reference list of phenolic compounds (including stilbenes) in grapevine (*Vitis vinifera* L.) roots, woods, canes, stems, and leaves. Antioxidants.

[35] Tang J., Dunshea F.R., Suleria H.A. (2020). Lc-esi-qtof/ms characterization of phenolic compounds from medicinal plants (hops and juniper berries) and their antioxidant activity. Foods.

[36] Kramberger K., Barlič-Maganja D., Bandelj D., Baruca Arbeiter A., Peeters K., Miklavčič Višnjevec A., Jenko Pražnikar Z. (2020). HPLC-DAD-ESI-QTOF-MS determination of bioactive compounds and antioxidant activity comparison of the hydroalcoholic and water extracts from two *Helichrysum italicum* species. Metabolites.

[37] Luo Y., Wang J., Li S., Wu Y., Wang Z., Chen S., Chen H. (2022). Discovery and identification of potential anti-melanogenic active constituents of Bletilla striata by zebrafish model and molecular docking. BMC Complement. Med. Ther..

[38] Rodrigues M.V.N., Vedovello A., Rodrigues R.A.F., Montanari Junior I., Rehder V.L.G. (2013). Improved method to obtain pfaffic acid as a marker for quality control. Quím. Nova.

[39] Liu M., Zhao S., Wang Y., Liu T., Li S., Wang H., Tu P. (2015). Identification of multiple constituents in Chinese medicinal prescription Shensong Yangxin capsule by ultra-fast liquid chromatography combined with quadrupole time-of-flight mass spectrometry. J. Chromatogr. Sci..

[40] Sun L., Tao S., Zhang S. (2019). Characterization and quantification of polyphenols and triterpenoids in thinned young fruits of ten pear varieties by UPLC-Q TRAP-MS/MS. Molecules.

[41] Li X.Y., Shang R., Fu M.C., Fu Y. (2015). Conversion of biomass-derived fatty acids and derivatives into hydrocarbons using a metal-free hydrodeoxygenation process. Green Chem..

[42] El-Askary H., Handoussa H., Badria F., El-Khatib A.H., Alsayari A., Linscheid M.W., Abdel A., Motaal A. (2019). Characterization of hepatoprotective metabolites from *Artemisia annua* and *Cleome droserifolia* using HPLC/PDA/ESI/MS–MS. Rev. Bras. Farmacogn..

[43] Yan G., Zou D., Zhang A., Tan Y., Sun H., Wang X. (2015). UPLC-Q-TOF-MS/MS fingerprinting for rapid identification of the chemical constituents of *Ermiao Wan*. Anal. Methods.

[44] Zhang Y.D., Li P., Zheng N., Jia Z.W., Meruva N., Ladak A., Cleland G., Wen F., Li S.L., Zhao S.G., Wang J.Q. (2018). A metabolomics approach to characterize raw, pasteurized, and ultra-high temperature milk using ultra-performance liquid chromatography–quadrupole time-of-flight mass spectrometry and multivariate data analysis. J. Dairy Sci..

[45] Liu Y., Abreu P. (2006). Tirucallane triterpenes from the roots of *Ozoroa insignis*. Phytochemistry (Elsevier).

[46] Reidah I.M.A., Ibrahim M. (2013).

[47] Kitajima J., Kamoshita A., Ishikawa T., Takano A., Fukuda T., Isoda S., Ida Y. (2003). Glycosides of *Atractylodes lancea*. Chem. Pharm. Bull..

[48] Sun Y., Feng G., Zheng Y., Liu S., Zhang Y., Pi Z., Song F., Liu Z. (2020). Putative multiple reaction monitoring strategy for the comparative pharmacokinetics of postoral administration Renshen–Yuanzhi compatibility through liquid chromatography–tandem mass spectrometry. J. Ginseng Res..

[49] Xu L.L., Guo F.X., Chi S.S., Wang Z.J., Jiang Y.Y., Liu B., Zhang J.Y. (2017). Rapid screening and identification of diterpenoids in *Tinospora sinensis* based on high-performance liquid chromatography coupled with linear ion trap-Orbitrap mass spectrometry. Molecules.

[50] Zhang J.Q., Yang M., Jiang B.H., Huang H.L., Chen G.T., Lu Z.Q., Li X.N., Bi K.S., Guo D.A. (2008). Analysis of major chemical constituents in Luan-Pao-Prescription using liquid chromatography coupled with electrospray ionization mass spectrometry. Nat. Prod. Commun..

[51] Zhang Y., Xiong H., Xu X., Xue X.U., Liu M., Xu S., Liu H., Gao Y., Zhang H., Li X. (2018). Compounds identification in semen cuscutae by ultra-high-performance liquid chromatography (UPLCs) couple to electrospray ionization mass spectrometry. Molecules.

