# Supplementary material for: Effects of seasonal variation on phytochemicals contributing to the antimalarial and antitrypanosomal activities of Breonadia salicina using a metabolomic approach
Source: Heliyon. 2024 Jan 13;10(2):e24068. doi: 10.1016/j.heliyon.2024.e24068 (PMC10827688; doi:10.1016/j.heliyon.2024.e24068)
Supplement: Multimedia component 1 [file mmc1.docx]

*Supplementary material*

**Effects of Seasonal Variation on Phytochemicals Contributing to the Antimalarial and Antitrypanosomal Activities of *Breonadia salicina* using a Metabolomic Approach**

**Dorcas Tlhapi ^a,*^, Isaiah Ramaite** **^a^, Chinedu Anokwuru ^b^, Teunis van Ree ^a^, Ntakadzeni Madala** **^c^ and Heinrich Hoppe ^d^**

^a^Department of Chemistry, Faculty of Science, Engineering and Agriculture, University of Venda, Private Bag X5050, Thohoyandou 0950, South Africa

^b^Department of Basic Sciences, School of Science and Technology, Babcock University, Nigeria

^c^Department of Biochemistry, Faculty of Science, Engineering and Agriculture, University of Venda, Private Bag X5050, Thohoyandou 0950, South Africa

^d^Department of Biochemistry and Microbiology, Rhodes University, Grahamstown 6140, South Africa

*Correspondence: E-mail: dorcastlhapi@gmail.com (D. Tlhapi)


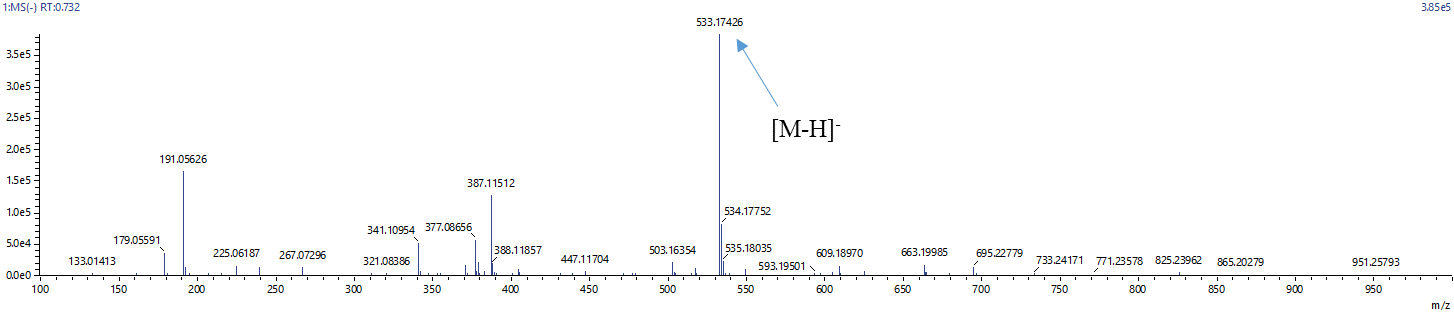


**Fig. S1**. Mass spectrum of quinic acid diglucoside.


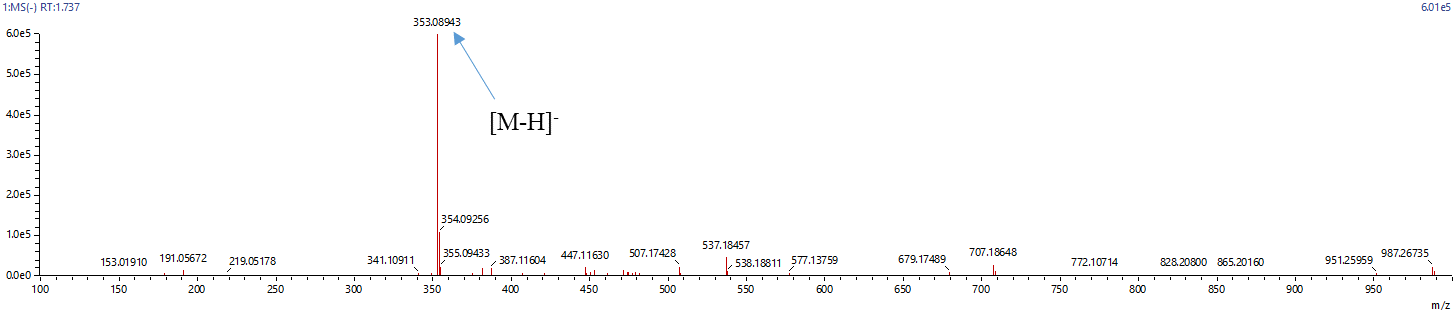


**Fig. S2**. Mass spectrum of chlorogenic acid [3,4-dihydroxycinnamoylquinic acid; 5-caffeoylquinic acid].


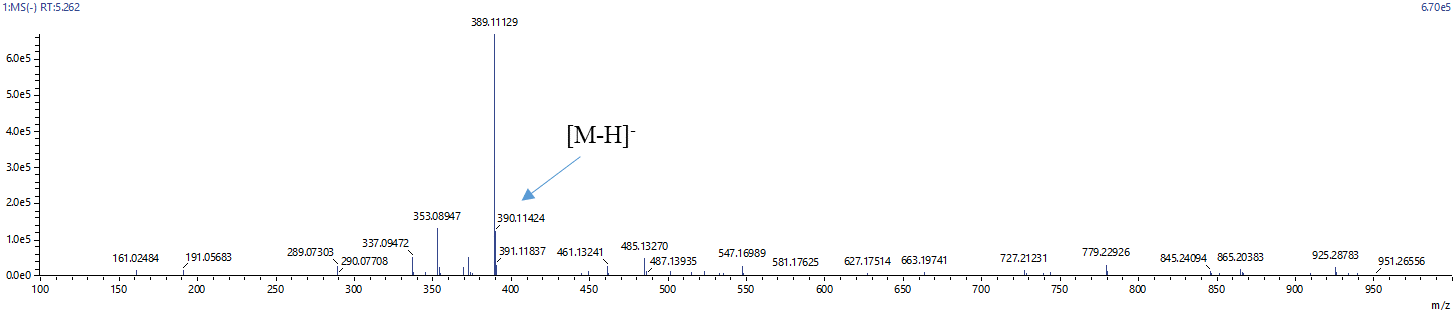


**Fig. S3**. Mass spectrum of *trans*-resveratroloside.


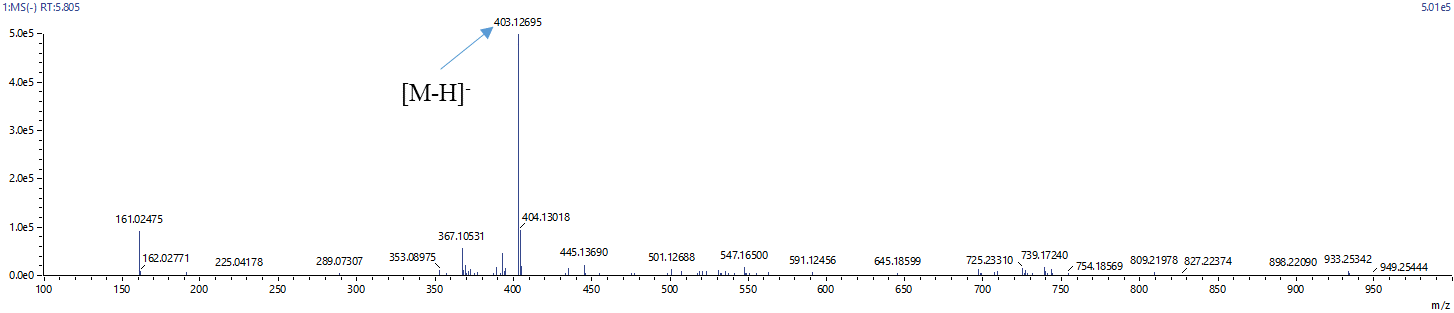


**Fig. S4**. Mass spectrum of oleoside 11-methylester.


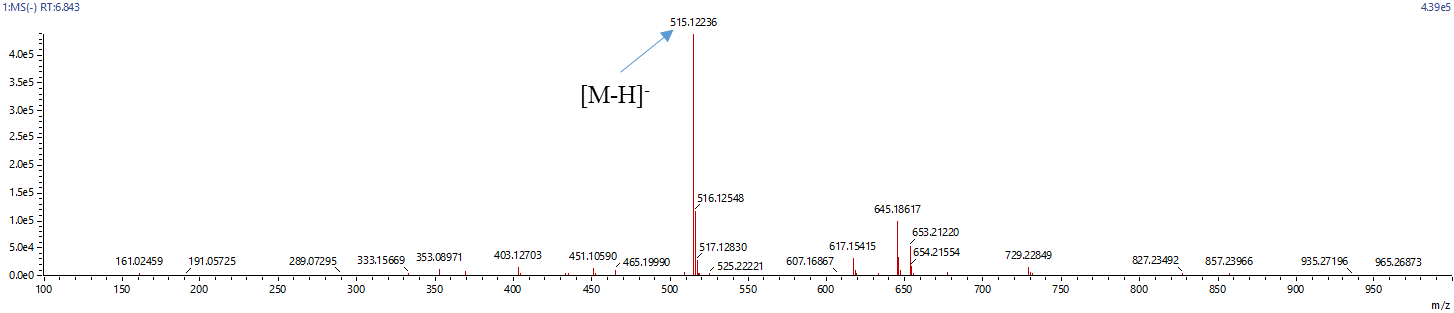


**Fig. S5**. Mass spectrum of dicaffeoyl quinic acid isomer: 3,4−diCQA.


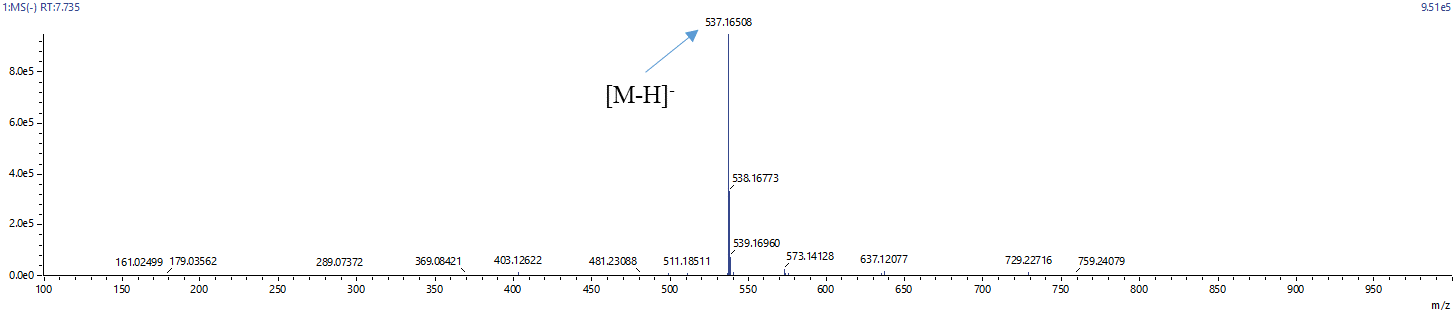


**Fig. S6.** Mass spectrum of 4,8,4’,8’-tetramethoxy-[1,1’- biphenanthrene]-2,7,2’,7’- tetrol.


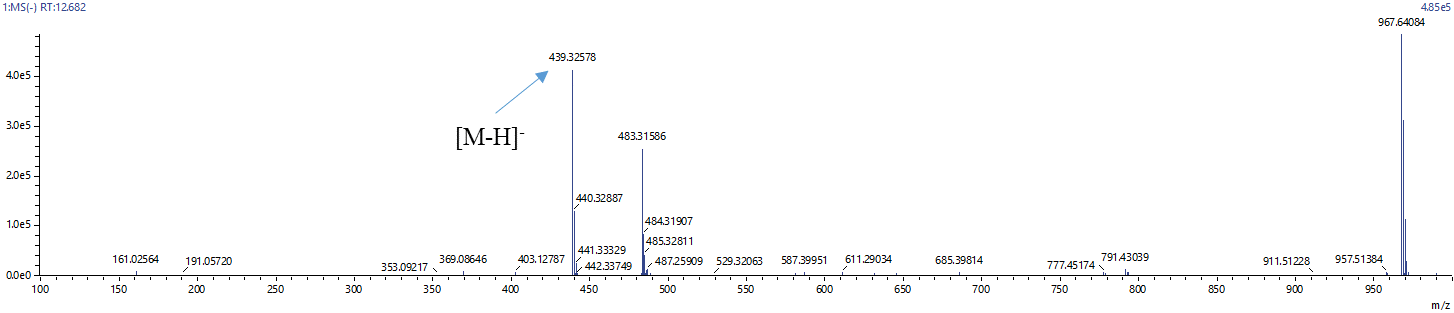


**Fig. S7**. Mass spectrum of pfaffic acid.


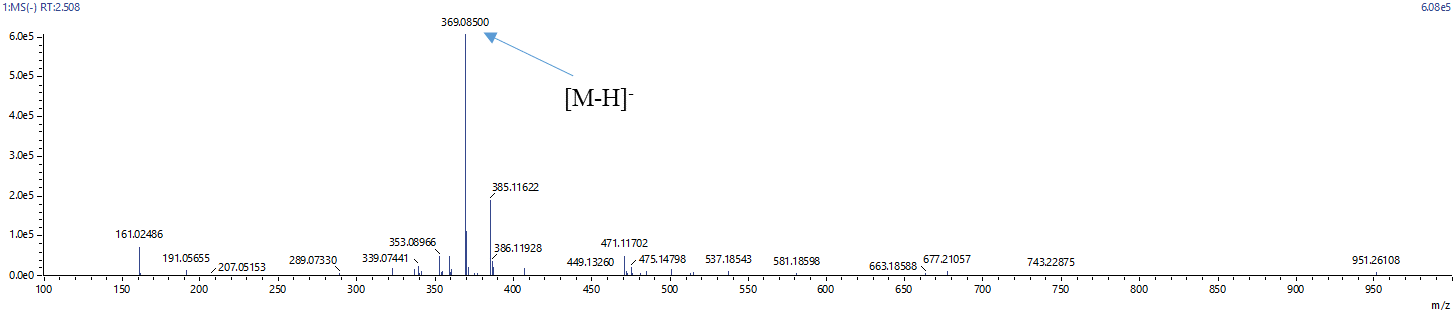


**Fig. S8**. Mass spectrum of ferulic acid 4-*O*-glucuronide.


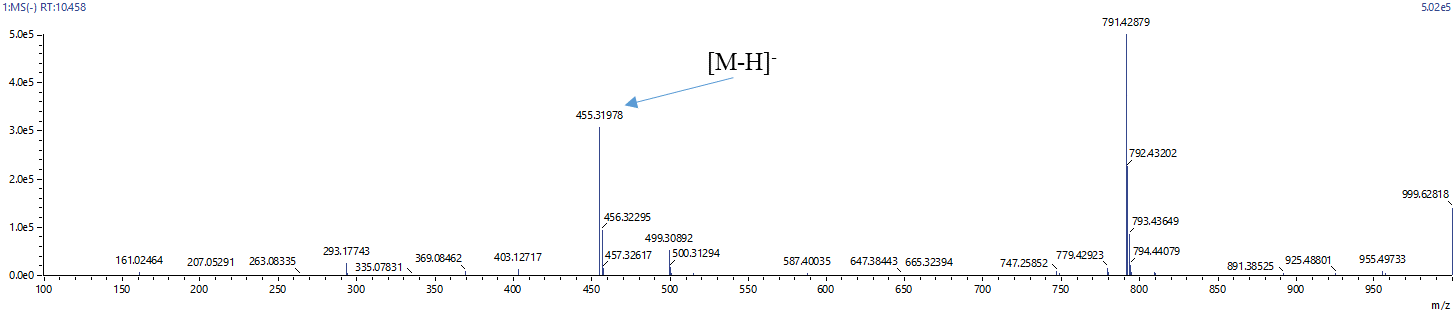


**Fig. S9**. Mass spectrum of ursolic acid.


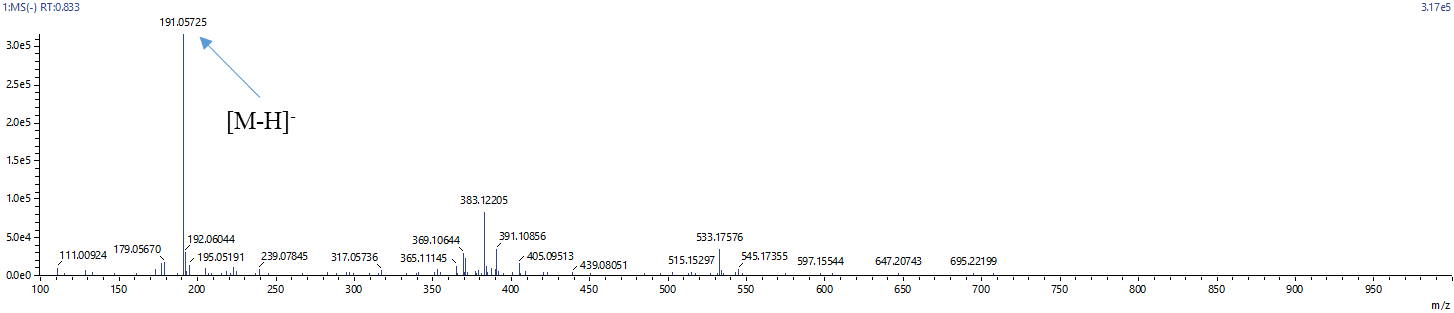


**Fig. S10.** Mass spectrum of quinic acid.


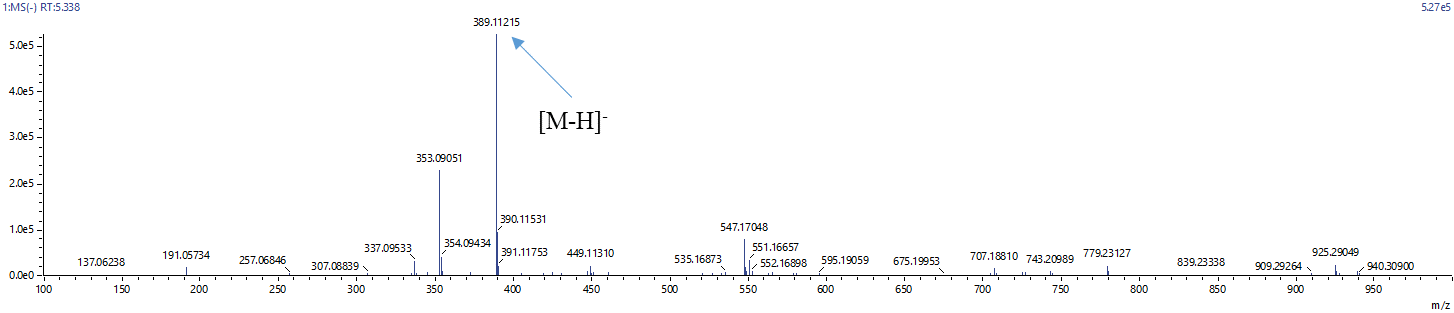


**Fig. S11**. Mass spectrum of deacetyl asperuloside acid.


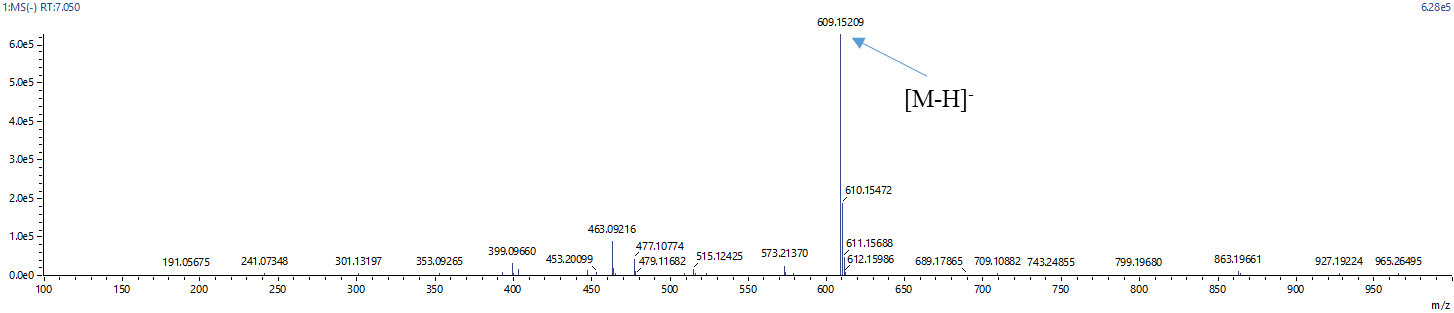


**Fig. S12.** Mass spectrum of rutin.


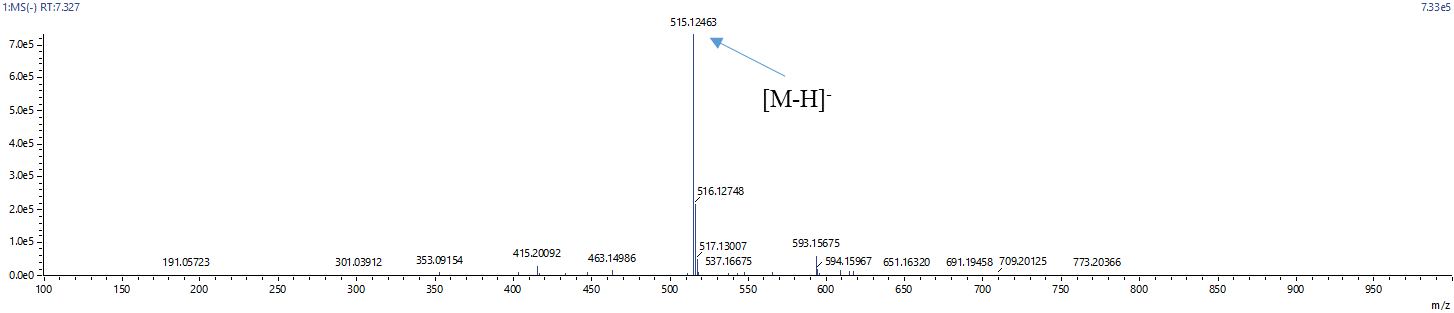


**Fig. S13**. Mass spectrum of di-*O*-caffeoylquinic acid.


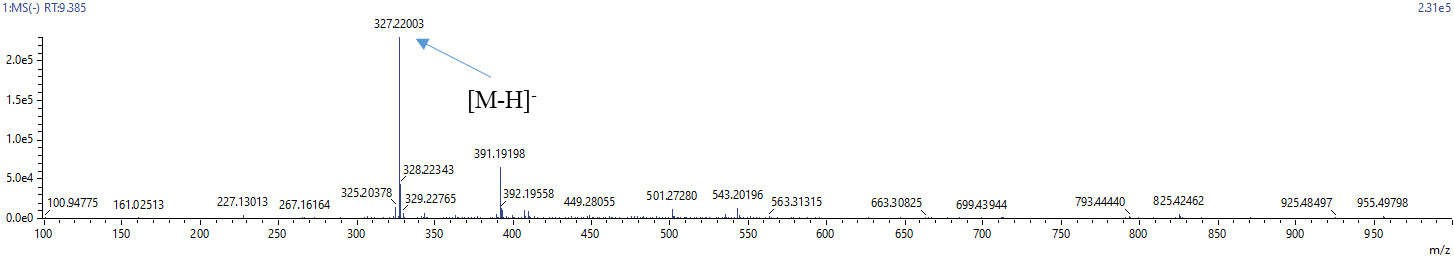


**Fig. S14**. Mass spectrum of trihydroxy-octadecadienoic acid.


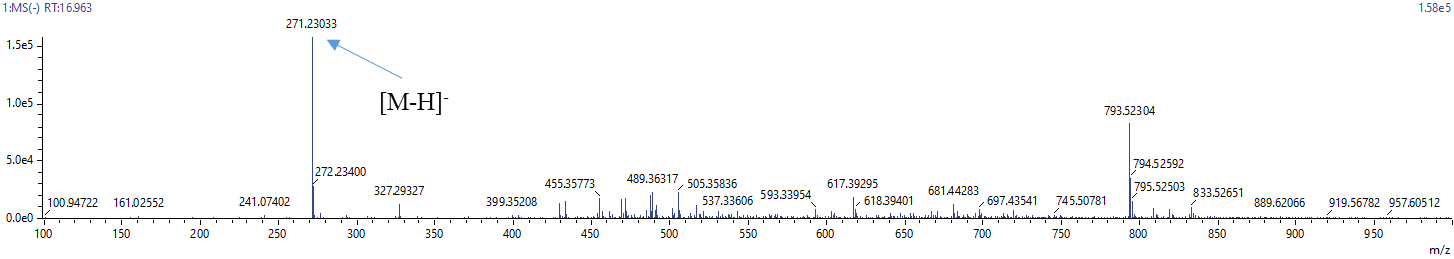


**Fig. S15**. Mass spectrum of 15-hydroxyhexadecanoic acid.


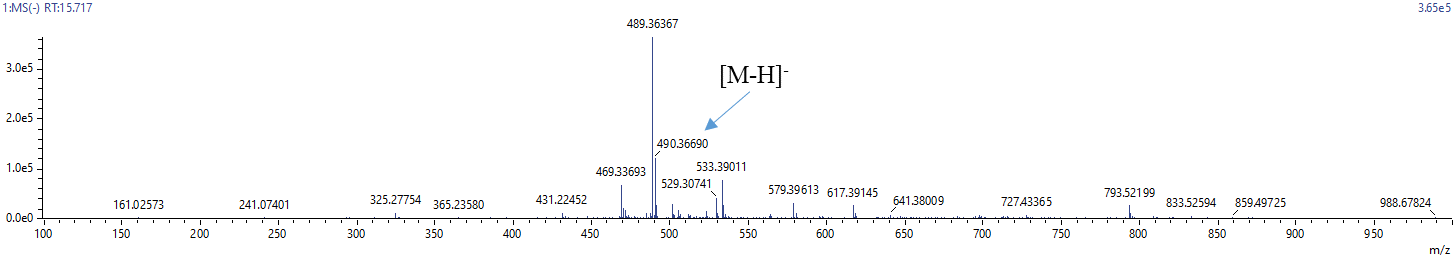


**Fig. S16**. Mass spectrum of 3-α,24R,25-trihydroxytirucall-8-en-21-oic acid.


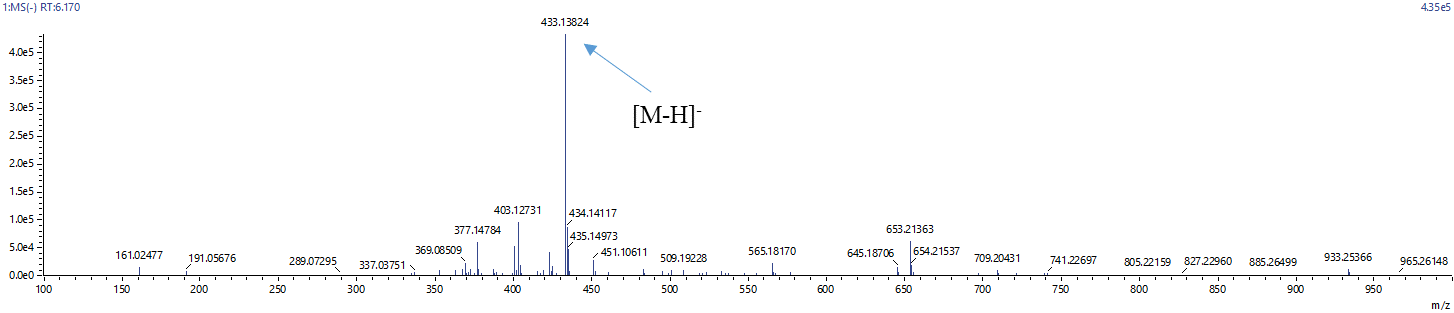


**Fig. S17**. Mass spectrum of 6-α-hydroxyforsythide dimethyl ester.


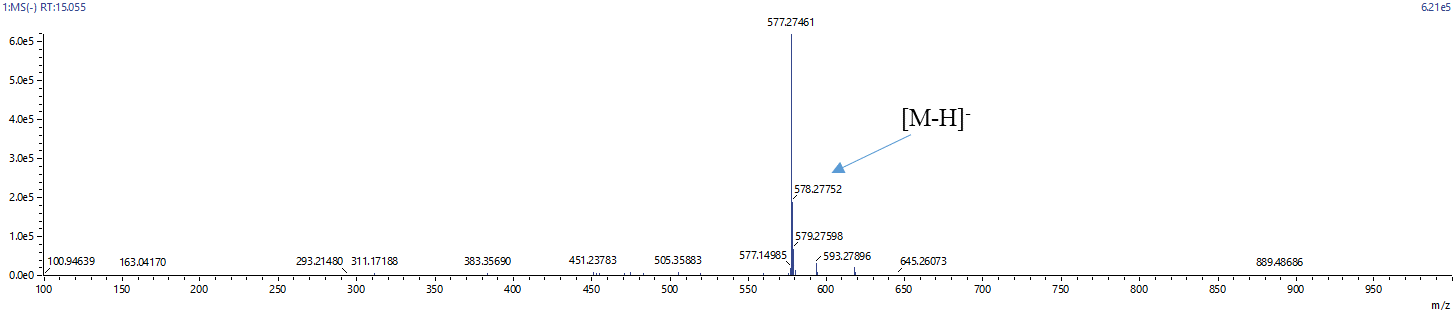


**Fig. S18**. Mass spectrum of atractyloside G 2-*O*-β-D-glucopyranoside.


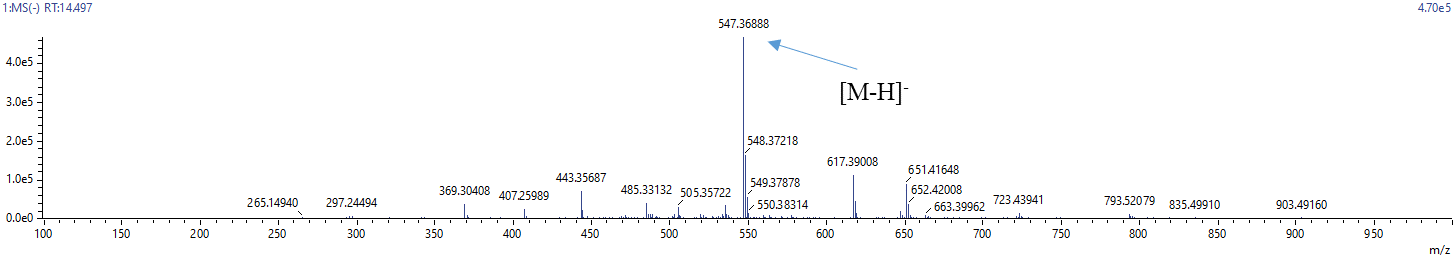


**Fig. S19**. Mass spectrum of sibiricose A6.


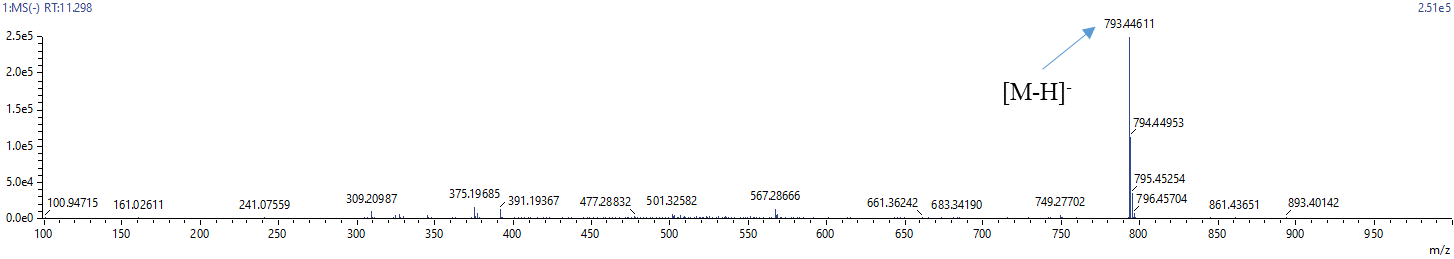


**Fig. S20**. Mass spectrum of zingibroside R1.


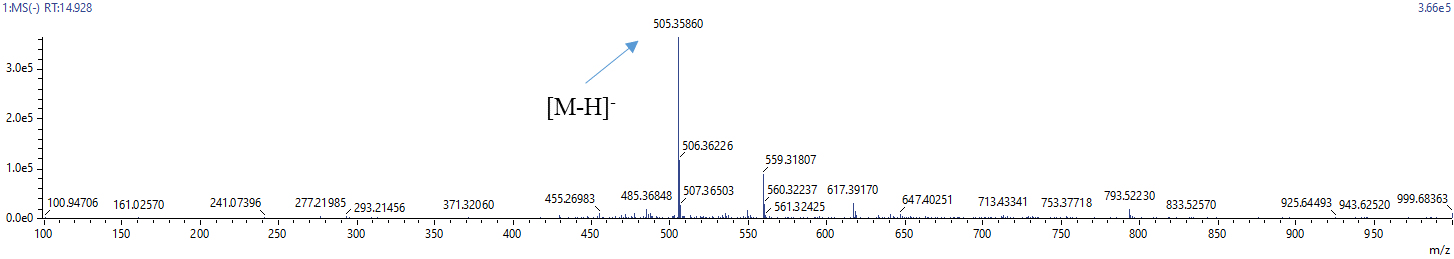


**Fig. S21**. Mass spectrum of isomers of quercetin 3-(2ʹʹ-acetylgalactoside).


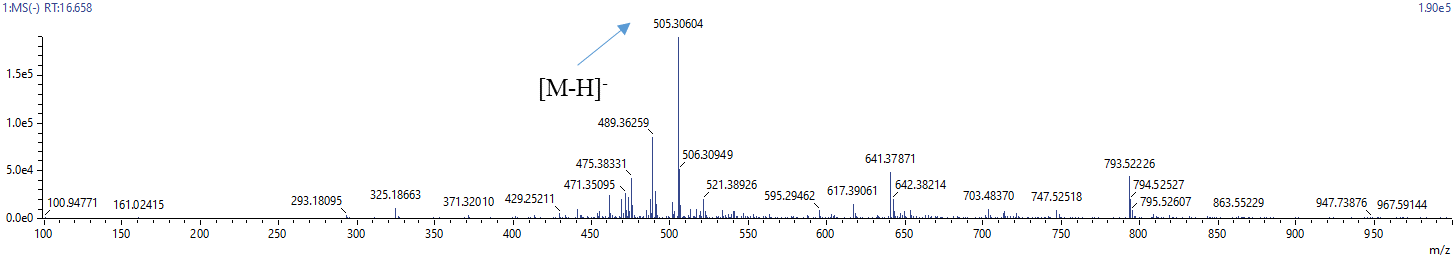


**Fig. S22**. Mass spectrum of isomers of tinospinoside B and tinospinoside C.


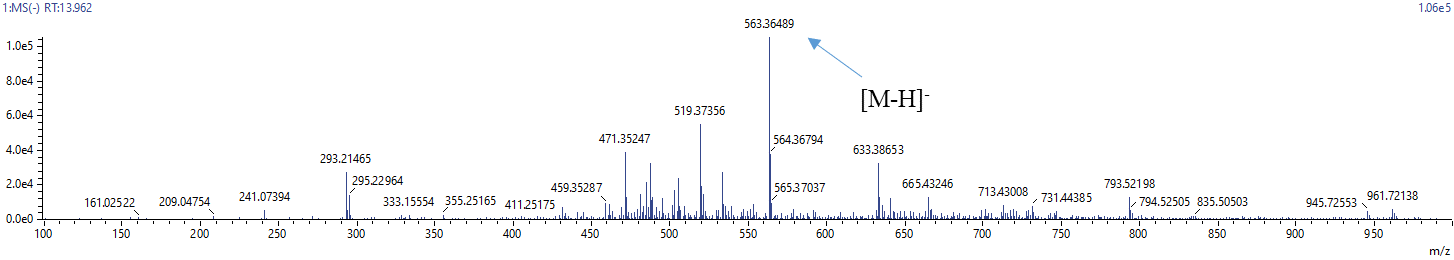


**Fig. S23**. Mass spectrum of apigenin-6-C-glu-8-C-ara.


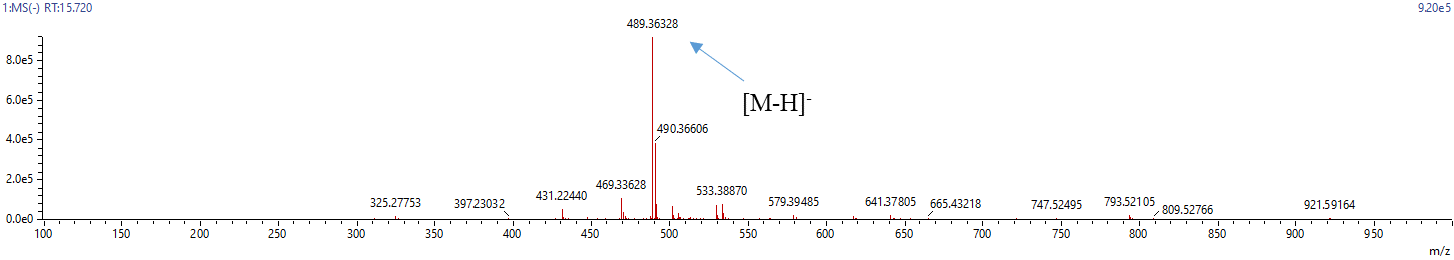


**Fig. S24**. Mass spectrum of isomers of 17-hydroxy-17-methyl-4- estrene-3-one 17-*O*-β-D-glucopyranoside.
